# The design strain sensitivity of the schenberg spherical resonant antenna for gravitational waves

**DOI:** 10.1038/s41598-023-43808-1

**Published:** 2023-10-17

**Authors:** V. Liccardo, C. H. Lenzi, R. M. Marinho, O. D. Aguiar, C. Frajuca, F. da Silva Bortoli, C. A. Costa

**Affiliations:** 1 Instituto Nacional de Pesquisas Espaciais, São José dos Campos, São Paulo 12227-010 Brazil; 2 Instituto Tecnológico de Aeronáutica, São José dos Campos, São Paulo 12228-900 Brazil; 3 Universidade Federal do Rio Grande, Rio Grande, Rio Grande do Sul 96203-900 Brazil; 4 Instituto Federal de São Paulo, São Paulo, São Paulo 01109-010 Brazil

**Keywords:** Astronomical instrumentation, Characterization and analytical techniques

## Abstract

The main purpose of this study is to review the Schenberg resonant antenna transfer function and to recalculate the antenna design strain sensitivity for gravitational waves. We consider the spherical antenna with six transducers in the semi dodecahedral configuration. When coupled to the antenna, the transducer-sphere system will work as a mass-spring system with three masses. The first one is the antenna effective mass for each quadrupole mode, the second one is the mass of the mechanical structure of the transducer first mechanical mode and the third one is the effective mass of the transducer membrane that makes one of the transducer microwave cavity walls. All the calculations are done for the degenerate (all the sphere quadrupole mode frequencies equal) and non-degenerate sphere cases. We have come to the conclusion that the “ultimate” sensitivity of an advanced version of Schenberg antenna (aSchenberg) is around the standard quantum limit (although the parametric transducers used could, in principle, surpass this limit). However, this sensitivity, in the frequency range where Schenberg operates, has already been achieved by the two aLIGOs in the O3 run, therefore, the only reasonable justification for remounting the Schenberg antenna and trying to place it in the sensitivity of the standard quantum limit would be to detect gravitational waves with another physical principle, different from the one used by laser interferometers. This other physical principle would be the absorption of the gravitational wave energy by a resonant mass like Schenberg.

## Introduction

Gravitational waves (GW) are ripples in the fabric of space-time generated by the acceleration of massive cosmic objects. These ripples move at the speed of light and can excite quadrupolar normal-modes of elastic bodies. The first detection of GWs from the inward spiral and merger of a pair of Black Holes (BH) (GW150914) has been widely discussed in the literature^1–4^. Furthermore, the recent simultaneous detection of the electromagnetic counterpart with GWs from a binary Neutron Star (NS) merger (GW170817) has officially begun the era of multi-messenger astronomy involving GWs^5,6^. Studying the universe with these two fundamentally different types of information will offer the possibility of a richer understanding of the astrophysical scenarios as well as of nuclear processes and nucleosynthesis. For the first time in the GW astronomy, it has been possible to determine the position in the sky of the source thanks to the detection, at the same time, of the three interferometers of the LIGO/Virgo collaboration^5^.

**Figure 1:**
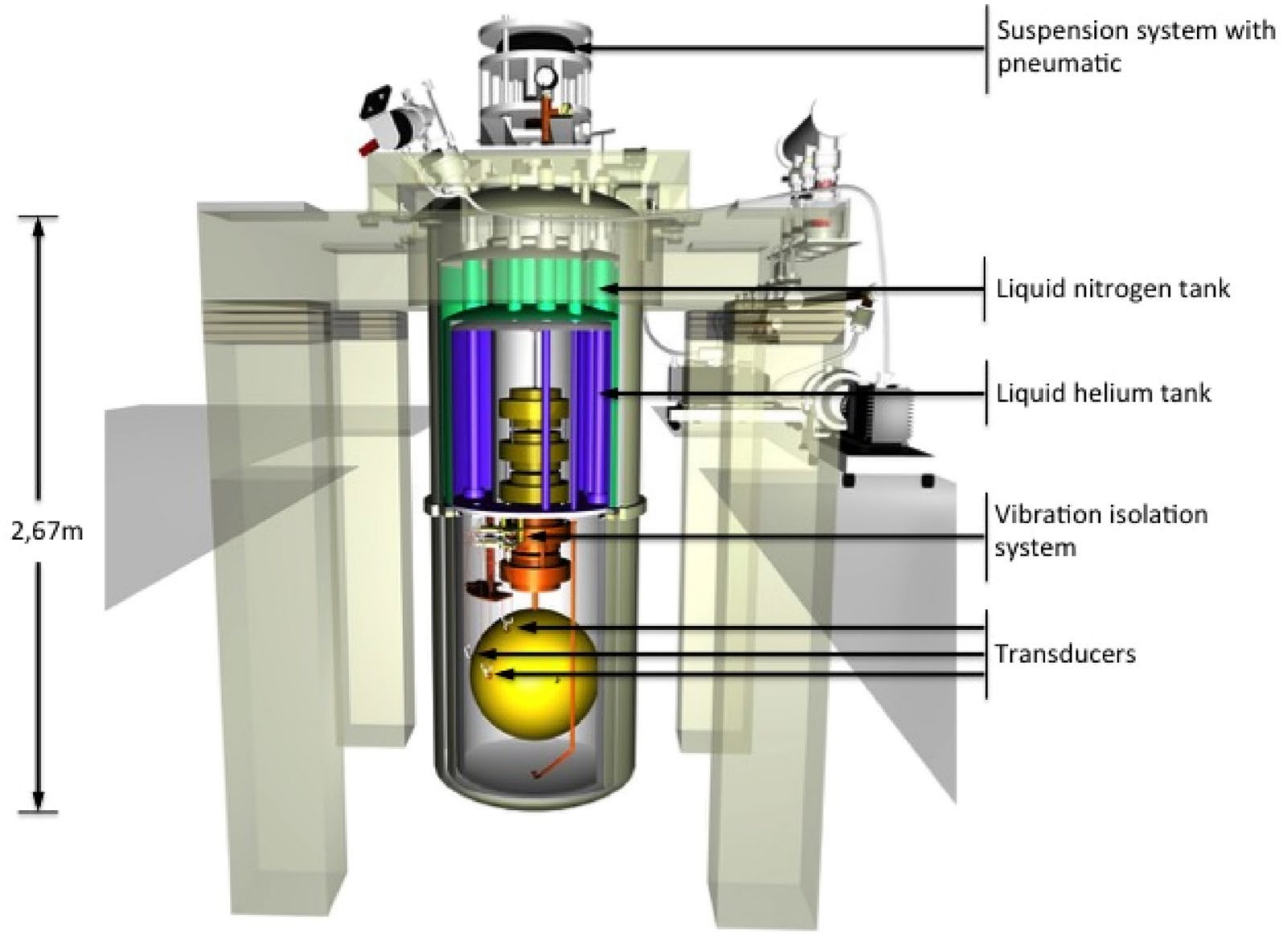
The Schenberg antenna where nine parametric transducers monitor the fundamental modes of vibration of the resonant spherical mass (credit: Xavier P. M. Gratens).

The Mario Schenberg Brazilian detector is based on the detection of five quadrupole modes relative to the mechanical vibrations of a spherical resonant-mass of $M_S= 1124$ kg and radius $R=32.33$ cm (Fig. 1). The operating frequency band is 3.15–3.26 kHz. The antenna is made of a CuAl(6%) alloy, which has a high mechanical quality factor Q $\sim$ $2\times 10^{6}$ at 4 K. The system is suspended by a vibration isolation system, capable of attenuating external vibrations by about 300 dB^7,8^. The instrument will be maintained at low temperatures ($\sim$ 4 K) by cryogenic chambers (dewars), cooled down by a He flow^9^. The antenna is coupled to parametric transducers that will monitor the vibrations of the quadrupolar/monopolar normal modes of the sphere^10–14^. One of the main advantages of a GW spherical resonant antenna is its omnidirectional sensitivity, which makes it equally responsive to all wave directions and polarizations^15^. Spherical resonant-mass antennas have been already intensively studied^16–18^. The designed antenna transduction system consists of nine transducers fixed on the surface of the sphere, six of which follow the truncated icosahedron configuration proposed by Johnson and Merkowitz^19^. This configuration presents some benefits and allows the simplification of the equations of motion, the determination of the GW direction in the sky, and facilitates the interpretation of the signal. For more details on the Schenberg antenna, the reader is referred to^20,21^ and references therein. It is important to mention that, in addition to being a device to try to detect gravitational waves, the Schenberg antenna could also be used to test the hypothesis that the ripples in the curvature of the fabric of space-time can be scaled by a more minute “action”, whose detection requires sensitivities beyond the standard quantum limit^22^. On the other hand, the Schenberg detector can also be used to test alternative theories of gravitation, such as the reference^23^ which, having a massive graviton, has six polarization states. The plan of the paper is as follows: in section “Gravitationalwaves from NS-BH binary systems”, we consider the emission of GWs from the spiraling of a NS-BH binary system and we discuss the detectability of this system by the Schenberg antenna. Then, we discuss the interaction of GWs with matter in section “The interaction of GW with matter”. The detector model is introduced in section “The detector model”, which is followed by the calculation of the response function of the antenna. All the calculations are done for the degenerate (all the sphere quadrupole mode frequencies equal) and non-degenerate sphere cases. For the degenerate case, the degenerate frequency was chosen as the average of the five quadrupole frequencies measured at 2 K. Final considerations as well as the discussion of the results are presented in section “Discussions and conclusions”.

## Gravitational waves from NS-BH binary systems

Coalescence of NS-BH binaries is one of the most promising GW sources for ground-based antennas. NS-BH systems are believed to be formed as a result of two supernovae in a massive binary system^24,25^. GWs from binaries involving NS represent a tool to study NS properties like the radius, compactness, and tidal deformability. Knowledge of NS properties will allow constraining the equation of state of nuclear-density matter^26^, giving us valuable information on nuclear physics. After the formation of the system, the orbital separation decreases gradually due to the long-term gravitational radiation reaction (i.e., two objects are in an adiabatic inspiral motion), and eventually, the two objects merge into a BH. The final fate of the binary depends primarily on the mass of the BH and the compactness of the NS. However, a detailed analysis has shown that the BH spin and the NS equation of state also play an important role in determining the final fate^25^. The effective-one-body (EOB) formalism was introduced^27,28^ as a promising approach to describe analytically the inspiral, merger, and ringdown waveforms emitted during a binary merger. Among the candidates of electromagnetic counterparts, a short-hard Gamma-Ray Burst (GRB) and its afterglow are vigorously studied both theoretically and observationally^29,30^. For a deeper analysis of NS-BH binaries, see^25^.

**Figure 2:**
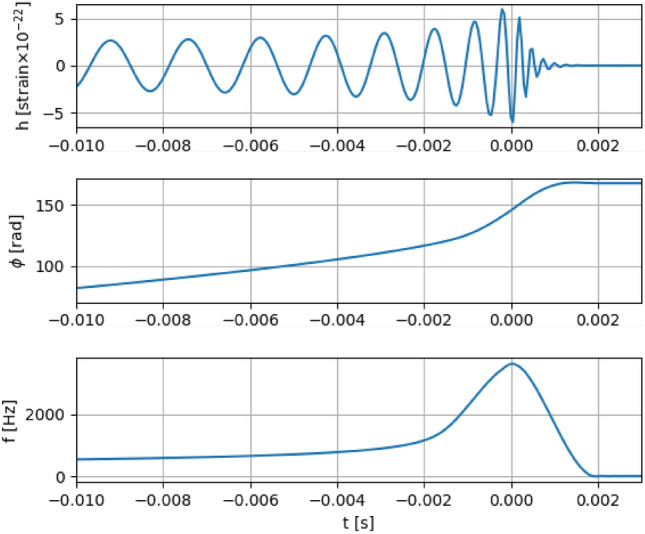
The GW strain signal produced by the coalescence of a non-spinning 1.4–3.0 $M_{\odot }$ NS-BH binary system (*Top*), phase (*Middle*) and frequency (*Bottom*) are plotted as function of the time before merging.

In this section, we discuss the GW signal produced by the coalescence of a non-spinning 1.4–3.0 $M_{\odot }$ NS-BH binary system, disregarding finite-size effects such as tidal deformation. The narrow frequency window of the antenna constrains the BH mass to be $\lesssim$ 3 $M_{\odot }$. Compact binary systems emit periodic GWs, whose frequencies sweep the spectrum until they reach their maximum values when they are close to the coalescence. The characteristic amplitude and the frequency of GWs near the last orbit are given by^25^

1 $$\begin{aligned}{} & {} h \approx 3.6 \times {10^{ - 22}}\left( {\frac{{{M_{BH}}}}{{6{M_ \odot }}}} \right) \left( {\frac{{{M_{NS}}}}{{1.4{M_ \odot }}}} \right) \left( {\frac{{6GM}}{{{c^2}r}}} \right) \left( {\frac{{0.1{\text{Gpc}}}}{D}} \right) , \end{aligned}$$

2 $$\begin{aligned}{} & {} f \approx \frac{\omega }{\pi } \approx 594 \, {\text{Hz}}{\left( {\frac{{6GM}}{{{c^2}r}}} \right) ^{\frac{3}{2}}}\left( {\frac{{7.4{M_ \odot }}}{M}} \right) , \end{aligned}$$

where $\omega$ is the angular velocity, *M* = $M_{BH}$ + $M_{NS}$, and *r* and *D* are the orbital separation and the distance to the source, respectively. The binary system studied may be in principle detected since the frequency of the gravitational signal $\sim$ 1 ms before coalescing falls in the band of the Brazilian antenna. NS-BH mergers are also potential targets of interferometers GW detectors. Since these kinds of antennas are sensitive in a much broader frequency range ($\sim$ 10–4000 Hz) they will detect the signal before the Schenberg antenna (during the inspiral phase). It is worth noting that due to the truncated icosahedron configuration the antenna is able to determine the polarization and the position of astrophysical sources of the GW^31–34^. There are a large number of waveform families in the literature, obtained from considerations about the type of source and approximation procedures used for the simulation (numerical relativity (NR), EOB formalism, post-Newtonian (PN) approximation, etc.). The gravitational signal for our analysis was generated using the PyCBC software package^35,36^. The waveform employed is one of those that are used by LIGO/Virgo, that is, the effective-one-body model tuned to numerical relativity (EOBNRv2). PN results are good as long as the velocities of the objects are not extreme relativistic. However, as the two objects orbit around each other, they lose energy through the emission of GWs, and their distance shrinks along with an increase in velocity. Consequently, PN predictions become more and more inaccurate the closer the binary gets to the merger, while the EOB approach, close to the merger, provides better accuracy by calibrating higher-order vacuum terms to NR waveforms. The EOBNRv2 waveform is believed to be sufficiently accurate to search for signals from non-spinning coalescing compact binaries in the aLIGO sensitive band. The EOB formalism has been refined several times to incorporate additional information from NR. Depending on the number of available NR waveforms as well as the modifications introduced to the EOB description, various versions of such EOBNR models have been developed^37,38^. It is beyond the scope of this paper to show the technical details of the EOB formalism and its extensions. Figure 2 also shows the waveform of the non-spinning NS-BH binary considered here. The waveform has also been re-sampled to be compatible with the sampling rate of the Schenberg antenna.

The coalescence rate of this type of system is very small and can be calculated indirectly. Upper limits ($\sim$ $10^{3}$ Gpc$^{-3}$ year$^{-1}$) were given assuming that all short GRBs/kilonovae are linked with NS-BH mergers^29^ and from the assumption that all the r-process material were produced in NS-BH coalescences^39^.

There are indications that NS-BH binary has been directly observed^40^ and an estimated rate density of $\sim$ 0.04 $\times$ $10^{3}$ Gpc$^{-3}$ year$^{-1}$ can also be derived from stellar evolution synthesis^41,42^. In the present work, to evaluate the event rate related to NS-BH mergers, we follow Li et al.^43^ and Abbott et al.^44^, who constrain the merger rate to be less than 6500 Gpc$^{-3}$ year$^{-1}$, assuming a population of binary systems of 1.4–3 $M_{\odot }$. This estimate is sensitive to physical parameters, such as the equation of state of NS material and the mass/spin distribution of the BH. The upper limit of the rate decreases for BHs with larger masses. The expected rates for other transient sources are smaller and/or less reliable. In order to be detected, the amplitude of the GW signal needs to be compatible with the sensitivity of the antenna.

For an advanced version of the Schenberg antenna (aSchenberg), which would operate around the standard quantum limit (section “The detector model”), gravitational signals with amplitude *h* $\sim$ 10$^{-22}$ could be detected at the nominal frequency of the antenna. In this case, a signal could be produced in GWs whose characteristic amplitude is $\sim$ 3 $\times$ 10$^{-22}$ at distances of the order of 0.1 Gpc (Fig. 2). In this volume, the event rate would be $\sim$ 3.6 year$^{-1}$ at a SNR $\sim$ 1. This conclusion relies on the validity of the assumption that all observed kilonovae were associated with NS-BH coalescences. In addition, many statistical studies based on the stellar evolution synthesis and supernova rates predict the rates at which NS-BH merge in the Milky Way and the nearby universe, assuming that Milky Way-like galaxies dominate, to be 1–10% of that of NS-NS binaries (every $\sim$ $10^{6}$–$10^{7}$ years)^45–47^. If we consider the contribution of elliptic galaxies the total coalescence rate of the universe could be increased by a significant fraction^48^. These estimates show that the prospect for the detection of NS-BH mergers of 1.4–3.0 $M_{\odot }$ by the Schenberg antenna can be very promising.

## The interaction of GW with matter

As it is well known, a GW produces a tidal density force at time *t* and at position ${{\varvec{x}}}$ given by (sum over repeated indices implied)

3 $$\begin{aligned} f_i^{GW}({{\varvec{x}}},t)=\frac{1}{2}\rho \ddot{h}_{ij}(t)x_j, \end{aligned}$$

where $\rho$ is the mass density and $\ddot{h}$ the second time derivative of the GW amplitude. Since the Schenberg antenna has a resonant frequency about 3 kHz, the wavelength of the GW detectable is about 100 km so we can use the value of $h_{ij}(t)$ at the center of the sphere. Equation (3) can be written in terms of the gradient of a potential

4 $$\begin{aligned} {{\varvec{f}}}^{GW}({{\varvec{x}}},t)=-\varvec{\nabla }\Phi ({{\varvec{x}}},t), \end{aligned}$$

where

5 $$\begin{aligned} \Phi ({{\varvec{x}}},t)= -\frac{1}{4}\rho x_i\ddot{h}_{ij}(t)x_j=-\frac{1}{4}\rho r^2 n_i\ddot{h}_{ij}(t)n_j, \end{aligned}$$

where ${\varvec{n}}$ is the unit vector in the radial direction and *r* the magnitude. We can expand $\Phi ({\varvec{x}},t)$ in terms of the real spherical harmonics, always used in this paper, $Y_{\ell m}^\mathcal{R}(\vartheta ,\varphi )$, defined in terms of the traditional spherical harmonics

6 $$\begin{aligned} Y_{\ell ,-m}^\mathcal{R}(\vartheta ,\varphi )&=\sqrt{2}\mathcal{I}[Y_{\ell m}(\vartheta ,\varphi )]\nonumber \\ Y_{\ell 0}^\mathcal{R}&= Y_{\ell 0} \nonumber \\ Y_{\ell m}^\mathcal{R}(\vartheta ,\varphi )&=\sqrt{2}\mathcal{R}[Y_{\ell m}(\vartheta ,\varphi )]. \end{aligned}$$

The spherical harmonics obey the normalization condition

7 $$\begin{aligned} \int _{\vartheta =0}^\pi \int _{\varphi =0}^{2\pi } Y_{\ell m}^\mathcal{R}Y_{\ell ' m'}^\mathcal{R} \sin \vartheta d\vartheta d\varphi =\delta _{\ell \ell '}\delta _{mm'}. \end{aligned}$$

From now on we will omit the superscript $\mathcal R$ and write $Y_{\ell m}^\mathcal{R}=Y_{\ell m}$. After the expansion we have (only terms with $\ell =2$, quadrupolar modes, survive)

8 $$\begin{aligned} \Phi ({\varvec{x}}, t) = -\sqrt{\frac{\pi }{15}}\rho r^2 \ddot{h}_m(t)Y_{2m}, \end{aligned}$$

where the $h_m$ are the expansion coefficients so called spherical amplitudes given by

9 $$\begin{aligned} h_{-2}&=h_{12} \end{aligned}$$

10 $$\begin{aligned} h_{-1}&=h_{23} \end{aligned}$$

11 $$\begin{aligned} h_0&=\frac{\sqrt{3}}{2}h_{33} \end{aligned}$$

12 $$\begin{aligned} h_1&=h_{13} \end{aligned}$$

13 $$\begin{aligned} h_2&=\frac{1}{2}(h_{11}-h_{22}). \end{aligned}$$

The spherical amplitudes $h_m$ for a GW coming from the direction defined by the polar and azimuthal angles $(\theta ,\phi )$ as seen from the lab frame is given by (see Appendix D):

14 $$\begin{aligned} h_{-2}&=\frac{1}{2}(1+\cos ^2\theta )\sin 2\phi h_++\cos \theta \cos 2\phi h_\times \end{aligned}$$

15 $$\begin{aligned} h_{-1}&=-\frac{1}{2}\sin 2\theta \sin \phi h_+-\sin \theta \cos \phi h_\times \end{aligned}$$

16 $$\begin{aligned} h_0&=\frac{\sqrt{3}}{2}\sin ^2\theta h_+ \end{aligned}$$

17 $$\begin{aligned} h_1&=-\frac{1}{2}\sin 2\theta \cos \phi h_++\sin \theta \sin \phi h_\times \end{aligned}$$

18 $$\begin{aligned} h_2&=\frac{1}{2}(1+\cos ^2\theta )\cos 2\phi h_+-\cos \theta \sin 2\phi h_\times . \end{aligned}$$

In matrix notation and after making the rotation around the polarization angle $\psi$, we have

19 $$  \left( {\begin{array}{*{20}l}    {h_{{ - 2}} }  \\    {h_{{ - 1}} }  \\    {h_{0} }  \\    {h_{1} }  \\    {h_{2} }  \\   \end{array} } \right) = \left( {\begin{array}{*{20}l}    {\frac{1}{2}(1 + \cos ^{2} \theta )\sin 2\phi } & {\cos \theta \cos 2\phi }  \\    { - \frac{1}{2}\sin 2\theta \sin \phi } & { - \sin \theta \cos \phi }  \\    {\frac{{\sqrt 3 }}{2}\sin ^{2} \theta } & 0  \\    { - \frac{1}{2}\sin 2\theta \cos \phi } & {\sin \theta \sin \phi }  \\    {\frac{1}{2}(1 + \cos ^{2} \theta )\cos 2\phi } & { - \cos \theta \sin 2\phi }  \\   \end{array} } \right)\left( {\begin{array}{*{20}c}    {\cos 2\psi } & { - \sin 2\psi }  \\    {\sin 2\psi } & {\cos 2\psi }  \\   \end{array} } \right)\left( {\begin{array}{*{20}l}    {h_{ + } }  \\    {h_{ \times } }  \\   \end{array} } \right),$$

which in a more compact form becomes,

20 $$\begin{aligned} \varvec{h}_m=\varvec{T}_{\textbf{v}}{\varvec{h}}. \end{aligned}$$

Using Eq. (8) and the vector spherical harmonics (see Appendix C we obtain the expression of the GW density force

21 $$\begin{aligned} {\varvec{f}}^{GW}=\sqrt{\frac{4\pi }{15}}\rho r\ddot{h}_m(t) \left( {\varvec{Y}}_{2m}^L+\frac{\sqrt{6}}{2}{\varvec{Y}}_{2m}^E\right) . \end{aligned}$$

In the case where ${\varvec{f}}$ in the right hand side of Eq. (32) is only of GW origin, the overlap integral

22 $$\begin{aligned} f^{GW}_{n\ell m}=\int _V\varvec{\Psi }_{n\ell m}({\varvec{x}})\cdot {\varvec{f}}^{GW}({\varvec{x}},t)d^3x \end{aligned}$$

is the effective force on each mode of the sphere and

23 $$\begin{aligned} \varvec{\Psi }_{n\ell m}(\varvec{x})= A_{n\ell }(r)\varvec{Y}_{\ell m}^{L}(\theta ,\phi )+ B_{n\ell }(r)\sqrt{\ell (\ell +1)}\varvec{Y}_{\ell m}^{E}(\theta ,\phi ) \end{aligned}$$

are the eigenfunctions of the uncoupled sphere modes, Eq. (33), repeated here for convenience. After the integration over the angular part this integral reduces, in the case of Schenberg antenna, to

24 $$\begin{aligned} f^{GW}_{n2m}=\frac{1}{2}\ddot{h}_m(t)M_SR\sqrt{\frac{3}{5\pi }}\int _0^1 \xi ^3(A_{n2}(\xi R)+3B_{n2}(\xi R))d\xi =\frac{1}{2}\ddot{h}_m(t)M_S\chi _n R, \end{aligned}$$

where

25 $$\begin{aligned} \chi _n=\sqrt{\frac{3}{5\pi }}\int _0^1 \xi ^3(A_{n2}(\xi R)+3B_{n2}(\xi R))d\xi . \end{aligned}$$

For the Schenberg antenna we have $\chi _1=-0.6004$.

We have calculated $\chi _1=\chi$ using the expression

$$\begin{aligned} \chi _1=\sqrt{\frac{3}{5\pi }}C_{12}\int _0^1\xi ^3(A_{12}(\xi R)+3B_{12}(\xi R))=-0.6004. \end{aligned}$$

Its worth mention that a similar expression is used by Maggiore^49^

$$\begin{aligned} \chi =\frac{3}{4\pi }C_{12}\int _0^1\xi ^3(A_{12}(\xi R)+3B_{12}(\xi R))=-0.32798, \end{aligned}$$

with the difference that in^49^ in the definition of the spherical amplitude $h_m$ is included the factor $\sqrt{16\pi /15}$.

## The detector model

As discussed above, the mechanical oscillations of the Schenberg antenna are monitored by a set of parametric transducers coupled on its surface. From a mathematical point of view, Johnson and Merkowitz^50^ proposed a model in which the output data from six transducers coupled to the antenna surface are related by decomposing them into the quadrupolar modes of the sphere. This method allows the reconstruction of the parameters that characterize the incident GW.

The movement equation for the displacement vector field ${\varvec{u}}({\varvec{x}},t)$ of a solid subjected to external forces density ${\varvec{f}}({\varvec{x}},t)$ is given by^51^

26 $$\begin{aligned} \rho \frac{\partial ^2{\varvec{u}}}{\partial t^2}- (\lambda _L+\mu _L)\varvec{\nabla }({\varvec{\nabla }\varvec{\cdot }\varvec{u}})- \mu _L\varvec{\nabla }^2{\varvec{u}}={\varvec{f}}, \end{aligned}$$

where $\lambda _L$ and $\mu _L$ are the tangential and volumetric Lamé coefficients of the material respectively. The initial conditions are ${\varvec{u}}({\varvec{x}},0)=0$ and $\dot{\varvec{u}}({\varvec{x}},0)=0$. The solution of (26) is obtained expanding the displacement vector ${\varvec{u}}({\varvec{x}},t)$ in series of the eigenfunctions $\varvec{\Psi }_N({\varvec{x}})$ of the equation

27 $$\begin{aligned} (\lambda _L+\mu _L)\varvec{\nabla }(\varvec{\nabla \cdot \Psi }({\varvec{x}}))+ \mu _L\varvec{\nabla }^2\varvec{\Psi }({\varvec{x}}) = -\rho \omega ^2\varvec{\Psi }({\varvec{x}}) \end{aligned}$$

subjected to the boundary condition of tension free at the surface of the sphere^49^

28 $$\begin{aligned} \lambda _L(\varvec{\nabla \cdot u})\varvec{{\hat{r}}}+ 2\mu _L(\varvec{{\hat{r}}\cdot \nabla }){\varvec{u}}+ \mu _L\varvec{{\hat{r}}\times }(\varvec{\nabla \times u})=0. \end{aligned}$$

The displacement vector field can be expanded as

29 $$\begin{aligned} {\varvec{u}}({\varvec{x}},t)=\sum _N a_N(t)\varvec{\Psi }_N({\varvec{x}}), \end{aligned}$$

where *N* is a set of indices, $a_N(t)$ is the time-dependent mode amplitude and $\varvec{\Psi }_N$ obeys the normalization condition

30 $$\begin{aligned} \int _V\rho ({\varvec{x}})\varvec{\Psi }_N({\varvec{x}})\cdot \varvec{\Psi }_{N'}({\varvec{x}})d^3x=M_S\delta _{NN'}. \end{aligned}$$

The integration is over the volume *V* of the sphere. After substituting (27) and (29) in (26), multiplying by $\varvec{\Psi }_{N'}$ and integrating over the volume of the sphere using (30), we obtain

31 $$\begin{aligned} M_S\ddot{a}_N(t)+\kappa _Sa_N(t)=\int _V\varvec{\Psi }_N({\varvec{x}})\cdot {\varvec{f}}({\varvec{x}},t)d^3x \end{aligned}$$

with $\kappa _S$ being the elastic constant.

At this point it is convenient to introduce a damping term in Eq. (31)

32 $$\begin{aligned} M_S\ddot{a}_N(t)+C_S\dot{a}_N(t)+\kappa _Sa_N(t)= \int _V\varvec{\Psi }_N({\varvec{x}})\cdot {\varvec{f}}({\varvec{x}},t)d^3x, \end{aligned}$$

where $C_S=w_N/Q_N$, $w_N$ the natural angular frequency of mode *N* and $Q_N$ the mechanical quality factor *Q* for mode *N*. The values of the parameters are given in Table (1).

### The uncoupled sphere

The solution of (27) subjected to the boundary condition of tension free at its surface are the natural modes of the sphere. They consist of two families of solution, the toroidal modes $\varvec{\Psi }_{n\ell m}^T$ and the spheroidal modes $\varvec{\Psi }_{n\ell m}$ (see^52^). We rewrite here this solution in terms of the vector spherical harmonics defined in Appendix C. Regarding the toroidal modes, in the case of a coupled sphere, they do not impart radial motion on the transducers, and the Schenberg detector is not sensitive to them, besides the fact that GWs do not excite these modes.

#### Spheroidal modes

The spheroidal modes are given by

33 $$\begin{aligned} \varvec{\Psi }_{n\ell m}(\varvec{x})= A_{n\ell }(r)\varvec{Y}_{\ell m}^{L}(\theta ,\phi )+ B_{n\ell }(r)\sqrt{\ell (\ell +1)}\varvec{Y}_{\ell m}^{E}(\theta ,\phi ), \end{aligned}$$

where

34 $$\begin{aligned} A_{n\ell }(r)&=C_{n\ell }\left[ \beta _{3}(k_{n\ell }R)j'_{\ell }(q_{n\ell }r)- \ell (\ell +1)\frac{q_{n\ell }}{k_{n\ell }}\beta _{1}(q_{n\ell }R) \frac{j_{\ell }(k_{n\ell }r)}{k_{n\ell }r} \right] \end{aligned}$$

35 $$\begin{aligned} B_{n\ell }(r)&=C_{n\ell }\left[ \beta _{3}(k_{n\ell }R)\frac{j_{\ell }(q_{n\ell }r)}{q_{n\ell }r}- \frac{q_{n\ell }}{k_{n\ell }}\beta _{1}(q_{n\ell }R)\beta _5(k_{n\ell }r)\right] . \end{aligned}$$

The transverse wave vectors $k_{n\ell }$, the longitudinal wave vectors $q_{n\ell }$ and the natural angular frequencies $w_{n\ell }=2\pi f_{n\ell }$ are the solution of the system of equations

36 $$\begin{aligned} \text{det}\left[ \begin{array}{cc} \beta _{4}(qR) &{} \ell (\ell +1)\beta _{1}(kR) \\ \beta _{1}(qR) &{} \beta _{3}(kR) \end{array}\right]&=0 \end{aligned}$$

37 $$\begin{aligned} qc_l&=w \end{aligned}$$

38 $$\begin{aligned} kc_t&= w, \end{aligned}$$

where betas are given by

39 $$\begin{aligned} \beta _{0}(z)&= \frac{j_{\ell }(z)}{z^{2}} \end{aligned}$$

40 $$\begin{aligned} \beta _{1}(z)&= \frac{d}{dz}\left( \frac{j_{\ell }(z)}{z}\right) \end{aligned}$$

41 $$\begin{aligned} \beta _{2}(z)&= \frac{d^2j_{\ell }(z)}{dz^2} \end{aligned}$$

42 $$\begin{aligned} \beta _{3}(z)&= \frac{1}{2}\beta _{2}(z)+ \left( \frac{\ell (\ell +1)}{2}-1\right) \beta _{0}(z) \end{aligned}$$

43 $$\begin{aligned} \beta _{4}(z)&= \beta _{2}(z)-\frac{\sigma }{1-2\sigma }j_{\ell }(z) \end{aligned}$$

44 $$\begin{aligned} \beta _{5}(z)&= \frac{1}{z}\frac{d}{dz}(zj_\ell (z)). \end{aligned}$$

The coefficients $c_l$ and $c_t$ are respectively the longitudinal

45 $$\begin{aligned} c_l=\sqrt{\frac{\mu _L}{\rho }}\sqrt{\frac{2-2\sigma }{1-2\sigma }} \end{aligned}$$

and transversal

46 $$\begin{aligned} c_t = \sqrt{\frac{\mu _L}{\rho }} \end{aligned}$$

velocities of the elastic waves. We define the ratio

47 $$\begin{aligned} \delta = \frac{c_l}{c_t}. \end{aligned}$$

here $\rho$ is the density of the sphere and $\sigma$ the Poisson ratio. The Poisson ratio can be written in terms of the ratio of the longitudinal and transversal sound velocities

48 $$\begin{aligned} \sigma = \frac{1}{2}\frac{\delta ^2-2}{\delta ^2-1}. \end{aligned}$$

The solution of the system of Eqs. (36–38) only depends on $c_l$ and $c_t$, in this way using the measured values of the monopole and quadrupole frequencies we were able to determine them. The results are given in Table 1.

The relationship between the Poisson ratio and the Young modulus *E* with the Lamé coefficients $\lambda _L$ and $\mu _L$ are

49 $$\begin{aligned} \frac{\lambda _L}{\mu _L}=\frac{2\sigma }{1-2\sigma }\quad \mu _L=\frac{E}{2(1+\sigma )}. \end{aligned}$$

### Antenna parameters at 4 K

The linear thermal expansion as a function of temperature is given by^53,54^

50 $$\begin{aligned} \alpha _\text{lin}(T)=\alpha _0\frac{\rho }{3BA}\left( \gamma c_V^\text{ion}(T)+\frac{2}{3} c_V^\text{el}(T) \right) , \end{aligned}$$

where $\alpha _0$ is a constant such that $\alpha _\text{lin}(273.15)=1.75\times 10^{-5}\,{\text{K}}^{-1}$^55^, *A* is the weighted average of CuAl6 atomic mass in kg, *B* is the bulk modulus

51 $$\begin{aligned} B = \frac{E}{3(1-2\sigma )}=\frac{2\rho c_t^2(1+\sigma )}{3(1-2\sigma )} \end{aligned}$$

and $\gamma$ is the weighted average of the CuAl6 Gruneisen coefficient. The lattice specific heat is

52 $$\begin{aligned} c_V^\text{ion}(T)=3R_Gf_D\left( \frac{\Theta _D}{T}\right) , \end{aligned}$$

where $R_G$ is the gas constant, $\Theta _D$ is the weight average of CuAl6 Debye’s temperature. The Debye’s function is

53 $$\begin{aligned} f_D(y)=\frac{3}{y^3}\int _0^y\frac{\text{e}^x x^4}{(\text{e}^x-1)^2}dx. \end{aligned}$$

The electrons specific heat is given by

54 $$\begin{aligned} c_V^\text{el}(T) = R_G\frac{\pi ^2}{2}\frac{T}{T_F} \end{aligned}$$

with $T_F$ being the weight average of CuAl6 Fermi temperature. Then the radius at 4 K will be given by

55 $$\begin{aligned} R=R_0+R_0\int _{300}^4\alpha _{lin}(T)dT. \end{aligned}$$

After calculating $c_l$ and $c_t$, based on its measured values at 300 K and 2 K and using the frequency of the monopolar mode and the mean frequency of the quadrupolar modes, we are able to calculate the radius of the sphere at 4 K. The solution must take into account that the coefficient of linear expansion depends on the Poisson’s ratio as well as the Eqs. (36–38) depends on it. With this methodology it is possible to calculate physical constants of CuAl6. The results are given in Table 1.

**Table 1:** Parameters of the Schenberg antenna.

**Description**	**Value**	**Method**
Quadrupole frequencies at 2 K	3172.485, 3183.000, 3213.623, 3222.900, 3240.000 ± 0.001Hz	Measured
Quadrupole frequencies at 300 K	3045, 3056, 3086, 3095, 3102 ± $0.5\,\text{Hz}$	Measured
Monopole frequency at 300 K	$f_{10}=6443.0\pm 0.5\,\text{Hz}$	Measured
Antenna’s radius at 300 K	$R_0=0.3233\,\text{m}$	Measured
Antenna mass	$M_S=1124\mathrm \, kg$	Measured
Antenna’s density at 300 K	$\rho =7938.523\pm 19\,{\text{kg}}/m^3$	Measured
Transducer first stage mass	$M_1=59.7100\pm 0.5\,{\text{mg}}$	Measured
Transducer second stage mass	$M_2=12.0\pm 0.5\, {\text{mg}}$	Measured
Monopole frequency at 4 K	$f_{10}=6713.42\,{\text{Hz}}$	Calculated
Mean quadrupole frequency at 4 K	${\bar{f}}_{12}=3205.94\,{\text{Hz}}$	Calculated
Longitudinal sound velocity at 4 K	$c_l=4937.6\,\text{m}/\text{s}$	Calculated using (36–38)
Transversal sound velocity at 4 K	$c_t=2448.2\,\text{m}/\text{s}$	Calculated using (36–38)
Linear thermal expansion coefficient at 273.15 K	$\alpha _0=1.75\times 10^{-5}\,\text{K}^{-1}$	Reference^55^
Weight average of CuAl6 Debye temperature	$\Theta _D=319.74\,\text{K}$	Reference^53^
Weight average of CuAl6 Fermi temperature	$T_F=84449.46\,\text{K}$	Reference^53^
Weight average CuAl6 Gruneisen coefficient	$\gamma =1.912$	Reference^56^
Sound velocities ratio	$r=2.016847$	Calculated using (47)
Poisson ratio	$\sigma =0.337010$	Calculated using (48)
Sphere radius as 4 K	$R = 0.32213\,\text{m}$	Calculated using (36–38, 55)
Sphere density at 4 K	$\rho =8025.04\,{\text{kg}}/\hbox {m}^3$	Calculated
Volumetric Lamé coefficient	$\mu _L=48.100\,{\text{GPa}}$	Calculated using (46)
Tangential Lamé coefficient	$\lambda _L=99.455\,{\text{GPa}}$	Calculated using (49)
Young modulus	$E = 128.621\,\text{GPa}$	Calculated using (49)
Bulk modulus	$B=131.522\,\text{GPa}$	Calculated using (51)
Chi factor	$\chi =-0.6004$	Calculated using (25)
Radial component factor at $r=R$	$\alpha =2.88345$	$\alpha =A_{12}(R)$
Antenna equivalent mass	$M_\text{eq}=340\,{\text{kg}}$	$M_\text{eq}= \frac{4\pi }{5\alpha ^2}M_S$
Antenna effective mass	$M_{\text{eff}}=283\,{\text{kg}}$	$M_{\text{eff}}=\frac{5}{6}M_\text{eq}$
Transducer amplification factor	$\text{amp}=\sqrt{\frac{M_{\text{eff}}}{M_2}}=4740$	

### The antenna coupled with transducers

**Figure 3:**
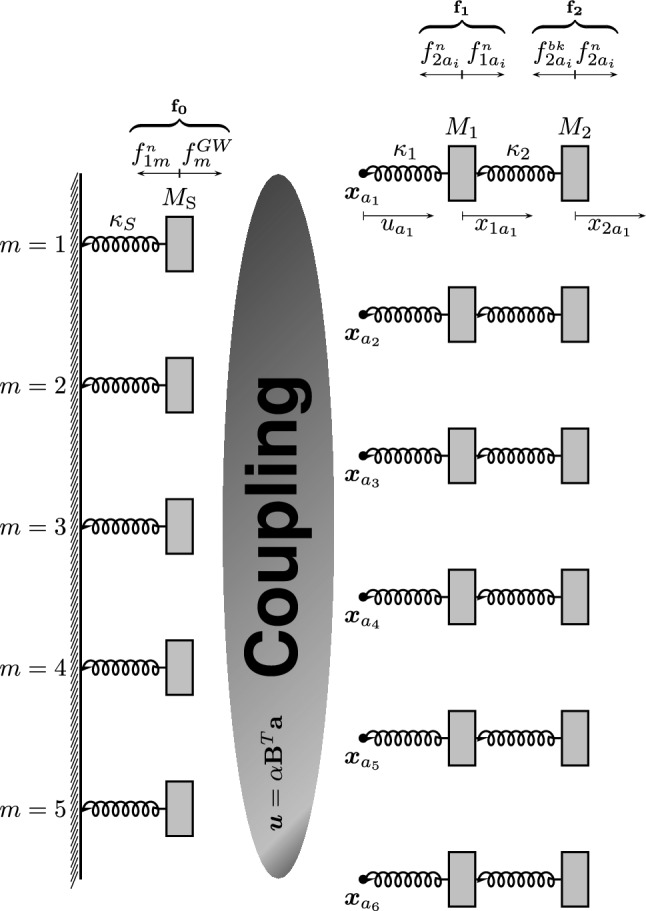
Schematic drawing representing in 2D the 3D coupling of the first five quadrupole (independent) modes of the Schenberg spherical antenna (left) with the six two-mode transducers (right). Each transducer more or less couples with each quadrupole mode of the sphere, depending on its position on the surface of the sphere in relation to the quadrupole mode in question. Due to these couplings, each transducer outputs information from all 17 modes. Only external forces and noises are represented in the figure. ${\varvec{f}}_0,\ {\varvec{f}}_1$ and ${\varvec{f}}_2$ are the resultant of all external forces and noises on the masses. Internal forces of action and reaction due to springs, ${\varvec{f}}^\kappa _i$ and ${\varvec{f}}^C_i$ are not represented in the figure.

In order to detect GWs, six two stage transducers are coupled to the Schenberg antenna^10^. Each stage of the transducers has the same resonance frequency of the first quadrupole mode $f_0=3205.94 \, \mathrm{Hz}$ (average of the five quadrupole frequencies measured at 2 K, also chosen as the degenerate frequency) and are sensitive only to the radial movement of the sphere. Transducers are devices that monitor the motion of the antenna surface. If a hypothetical GW excites the sphere quadrupolar modes, the corresponding mechanical energy will be transferred from the antenna to the transducers. Jonhson and Merkowitz^19^ discovered that if we use six transducers and locate each of them at the center of a pentagonal face of a truncated icosahedron projected onto one hemisphere of the sphere, then by a suitable linear combination of the output of the transducers, the so called mode channels, we can obtain a direct correspondence between the spherical amplitudes $h_m(t)$ of the GW and the quadrupolar modes of the sphere $a_{2m}(t)$. The angles of each of these transducers are given in Table 2. The Schenberg antenna makes use of two-modes parametric transducers. In this model the transducer motion is exclusively radial and only the *m* quadrupole modes are of interest. In an homogeneous sphere the modes are degenerated but in the real antenna they are not. The forces acting on the sphere (Fig. 3) are the GW force given by

**Table 2:** Polar and azimuthal angles $(\theta ,\phi )$ of the transducers positions, $\varphi =(1+\sqrt{5})/2$.

Transducer	$\theta$	$\phi$
T3	$\text{acos}\left( \frac{1}{\sqrt{3}\varphi \sqrt{\varphi +2}}\right) =79.18^{\circ }$	0°
T6	$\text{acos}\left( \frac{\varphi +1}{\sqrt{3}\sqrt{\varphi +2}}\right) = 37.37^{\circ }$	60°
T2	$\text{acos}\left( \frac{1}{\sqrt{3}\varphi \sqrt{\varphi +2}}\right) =79.18^{\circ }$	120°
T5	$\text{acos}\left( \frac{\varphi +1}{\sqrt{3}\sqrt{\varphi +2}}\right) = 37.37^{\circ }$	18°
T1	$\text{acos}\left( \frac{1}{\sqrt{3}\varphi \sqrt{\varphi +2}}\right) =79.18^{\circ }$	24°
T4	$\text{acos}\left( \frac{\varphi +1}{\sqrt{3}\sqrt{\varphi +2}}\right) = 37.377^{\circ }$	30°

56 $$\begin{aligned} {\varvec{f}}^{GW}=\sqrt{\frac{4\pi }{15}}\rho r\ddot{h}_m(t) \left( {\varvec{Y}}_{2m}^L+\frac{\sqrt{6}}{2}{\varvec{Y}}_{2m}^E\right) , \end{aligned}$$

the spring back reaction of the six transducers over the sphere at the positions ${\varvec{x}}_{a}$

57 $$\begin{aligned} {\varvec{f}}_1^\kappa =\sum _{a=1}^6\kappa _1(x_{1a}-u_a)\delta ({\varvec{x}}-{\varvec{x}}_a){{\varvec{e}}}_a, \end{aligned}$$

the damping back reaction of the resonators of the six transducers over the sphere at the positions ${\varvec{x}}_{a}$

58 $$\begin{aligned} {\varvec{f}}_1^C=\sum _{a=1}^6C_1(\dot{x}_{1a}-\dot{u}_a)\delta ({\varvec{x}}-{\varvec{x}}_a){{\varvec{e}}}_a, \end{aligned}$$

where $C_{1}$ is the damping term of the first resonator. The noise coming from the interaction with the resonator 1 is

59 $$\begin{aligned} {\varvec{f}}_1^{n}=\sum _{a=1}^6f_{1a}^{n}\delta ({\varvec{x}}-{\varvec{x}}_a){{\varvec{e}}}_a, \end{aligned}$$

where $x_{1a}$ is the displacement of the first resonator from its equilibrium position, ${{\varvec{e}}}_a$ is the radial unit vector at the position ${\varvec{x}}_a$ over the sphere and $u_a$ the deformation of the sphere at ${\varvec{x}}_a$ given by (repeated here for convenience)

60 $$\begin{aligned} u_a = \sum _{m=-2}^2a_m(t)\varvec{\Psi }_m({\varvec{x}}_a)\cdot {{\varvec{e}}}_a. \end{aligned}$$

The equation for $\varvec{\Psi }_m$, Eq. (33), is rewritten here with $n=1$, $\ell =2$, $A_{12}(r)=\alpha (r)$ and $B_{12}=\beta (r)$

61 $$\begin{aligned} \varvec{\Psi }_{m}(\varvec{x})= \alpha (r)\varvec{Y}_{m}^{L}(\theta ,\phi )+ \beta (r)\sqrt{6}\varvec{Y}_{m}^{E}(\theta ,\phi ), \end{aligned}$$

so that we have for $u_a$

62 $$\begin{aligned} u_a=\alpha (R)\sum _{m=-2}^2a_m(t)Y_m(\theta _a,\phi _a)= \alpha (R)\sum _{m=-2}^2a_m(t)B_{ma}. \end{aligned}$$

In matrix notation this is

63 $$\begin{aligned} \textbf{u}=\alpha \textbf{B}^T\textbf{a}, \end{aligned}$$

where $\alpha =\alpha (R)$ and the bold letters are matrices in which each entry of $\textbf{u}$ is related to a transducer and each entry of $\textbf{a}$ is related to a mode of the sphere. The movement equation for the displacement of the sphere surface $\textbf{u}$ is given in Appendix B.

The forces over the first resonator are the noise forces coming from the interaction of itself with the sphere, $f_1^{n}$, and with the second resonators, $f_2^{n}$, and the action and reaction of the restoration and damping forces of the first and second springs over it. The forces over the second resonator are the back action forces coming from the interaction with the microwave system, $f_2^{bk}$, the interaction with the resonator 1, $f_2^{n}$, and restoration and damping forces of the second springs over it, (Fig. 3).

The equations for the system are

64 $$\begin{aligned} M_S\ddot{a}_m(t)&= - C_S\dot{a}_m(t) -\kappa _S a(t) + \int \varvec{\Psi }_m({\varvec{x}})\cdot {\varvec{f}}({\varvec{x}},t)d^3x\end{aligned}$$

65 $$\begin{aligned} M_1\ddot{x}_{1a}&= f_{1a}^{n}-f_{2a}^{n} - \kappa _1(x_{1a}-u_a) - C_1(\dot{x}_{1a}-\dot{u}_a) + \kappa _2(x_{2a}-x_{1a}) + C_2(\dot{x}_{2a}-\dot{x}_{1a})\end{aligned}$$

66 $$\begin{aligned} M_2\ddot{x}_{2a}&= f_{2a}^{n} -f_{2a}^{bk}-\kappa _2(x_{2a}-x_{1a})- C_2(\dot{x}_{2a}-\dot{x}_{1a}), \end{aligned}$$

where ${\varvec{f}}({\varvec{x}},t)={\varvec{f}}_1^\kappa ({\varvec{x}},t)+{\varvec{f}}_1^C({\varvec{x}},t)-{\varvec{f}}_1^{n}({\varvec{x}},t)+{\varvec{f}}^{GW}({\varvec{x}},t)$ are the surface forces over the sphere and the GW force. The transducers frequencies are tuned with the frequency of the quadrupole mode of the homogeneous sphere $w_0$ such that

67 $$\begin{aligned} \frac{\kappa _S}{M_S}=\frac{\kappa _1}{M_1}=\frac{\kappa _2}{M_2}=w_0^2. \end{aligned}$$

For the real antenna we take $w_0$ as the mean value of the measured quadrupole mode frequencies $w_m$. For the maximum energy transfer from the sphere to the resonators the masses obeys the relation^57^

68 $$\begin{aligned} \frac{M_1}{M_{\text{eff}}}=\frac{M_2}{M_1}=\mu ^2\qquad \text {and}\qquad \frac{M_\text{S}}{M_{\text{eff}}}=\nu ^2, \end{aligned}$$

where the effective mass of the antenna $M_{\text{eff}}$ is calculated in the Appendix A. The integral in Eq. (64) can be written as

69 $$\begin{aligned} \int \varvec{\Psi }_m({\varvec{x}})\cdot {\varvec{f}}({\varvec{x}},t)d^3x =&\int \varvec{\Psi }_m\cdot {\varvec{f}}_1^\kappa d^3x+ \int \varvec{\Psi }_m\cdot {\varvec{f}}_1^Cd^3x\nonumber \\&- \int \varvec{\Psi }_m\cdot {\varvec{f}}_1^{n}d^3x+ \int \varvec{\Psi }_m\cdot {\varvec{f}}_m^{GW} d^3x. \end{aligned}$$

The first integral on the right hand side gives

70 $$\begin{aligned} \int \varvec{\Psi }_m\cdot {\varvec{f}}_1^\kappa d^3x=\kappa _1\alpha \sum _{a=1}^N Y_m(\theta _a,\phi _a)q_{1a}= \kappa _1\alpha \sum _{a=1}^N B_{ma}q_{1a}=\kappa _1\alpha [\textbf{B}{} \textbf{q}_1]_m. \end{aligned}$$

Similarly the second gives

71 $$\begin{aligned} \int \varvec{\Psi }_m\cdot {\varvec{f}}_1^C d^3x= C_1\alpha \sum _{a=1}^N Y_m(\theta _a,\phi _a)\dot{q}_{1a}= C_1\alpha [\textbf{B}{\dot{\textbf{q}}}_1]_m \end{aligned}$$

and the third

72 $$\begin{aligned} \int \varvec{\Psi }_m\cdot {\varvec{f}}_1^{n} d^3x= \alpha \sum _{a=1}^N Y_m(\theta _a,\phi _a)f_{1a}^{n}= \alpha [\textbf{B}{} \textbf{f}_1^{n}]_m, \end{aligned}$$

where $q_{1a}=x_{1a}-u_a$ and $q_{2a}=x_{2a}-x_{1a}$, the fourth is the Eq. (25). The result is

73 $$\begin{aligned} \int \varvec{\Psi }_m({\varvec{x}})\cdot {\varvec{f}}({\varvec{x}},t)d^3x= \kappa _1\alpha [\textbf{B}{} \textbf{q}_1]_m+ C_1\alpha [\textbf{B}{\dot{\textbf{q}}}_1]_m- \alpha [\textbf{B}{} \textbf{f}_1^{n}]_m+ {f}_m^{GW}(t). \end{aligned}$$

From now on we will use the column matrix

74 $$\begin{aligned} \textbf{f}^{GW}(t)=\left({\begin{array}{*{20}l} f_{-2}^{GW}(t) \\ f_{-1}^{GW}(t) \\ f_0^{GW}(t) \\ f_1^{GW}(t) \\ f_2^{GW}(t) \end{array}}\right). \end{aligned}$$

The equations in the new variables and in matrix notation are

75 $$\begin{aligned}&M_S{\ddot{\textbf{a}}} + C_S{\dot{\textbf{a}}}+\kappa _S\textbf{a}- C_1\alpha \textbf{B}{\dot{\textbf{q}}}_1 -\kappa _1\alpha \textbf{B}{} \textbf{q}_1 = \textbf{f}^{GW}- \alpha \textbf{B}{} \textbf{f}_1^{n}\nonumber \\&M_1\alpha \textbf{B}^T{\ddot{\textbf{a}}} +M_1{\ddot{\textbf{q}}}_1+ C_1{\dot{\textbf{q}}_{1}}-C_2{\dot{\textbf{q}}_{2}} + \kappa _1\textbf{q}_{1}-\kappa _2\textbf{q}_{2} = \textbf{f}_{1}^{n}- \textbf{f}_2^{n}\nonumber \\&M_2\alpha \textbf{B}^T{\ddot{\textbf{a}}} + M_2{\ddot{\textbf{q}}}_1+M_2{\ddot{\textbf{q}}}_2+ C_2{\dot{\textbf{q}}_{2}}+ \kappa _2\textbf{q}_{2} = \textbf{f}_{2}^{n}-\textbf{f}_2^{bk}. \end{aligned}$$

In block matrix notation we have

76 $$\begin{aligned}&\left[ \begin{array}{ccc} M_S\,\textbf{I} &{}\quad \textbf{0} &{}\quad \textbf{0} \\ M_1\alpha \textbf{B}^T &{}\quad M_1\textbf{I} &{}\quad \textbf{0} \\ M_2\alpha \textbf{B}^T &{}\quad M_2\textbf{I} &{}\quad M_2\textbf{I} \end{array}\right] \left[ \begin{array}{c} {\ddot {\mathbf{a}}}\\ {\ddot{\textbf{q}}}_1 \\ {\ddot {\mathbf{q}}}_2 \end{array}\right] + \left[ \begin{array}{ccc} {Diag}({C_m}_S) &{}\quad -C_1\alpha \textbf{B} &{}\quad \textbf{0} \\ \textbf{0} &{}\quad C_1\textbf{I} &{}\quad -C_2\textbf{I} \\ \textbf{0} &{}\quad \textbf{0} &{}\quad C_2\textbf{I} \end{array}\right] \left[ \begin{array}{c} {\dot{{\mathbf{a}}}}\\ {\dot{\textbf{q}}}_1 \\ {\dot {\mathbf{q}}}_2 \end{array}\right] \nonumber \\&\quad + \left[ \begin{array}{ccc} \text{Diag}({\kappa _m}_S) &{}\quad -\kappa _1\alpha \textbf{B} &{}\quad \textbf{0} \\ \textbf{0} &{}\quad \kappa _1\textbf{I} &{}\quad -\kappa _2\textbf{I} \\ \textbf{0} &{}\quad \textbf{0} &{}\quad \kappa _2\textbf{I} \end{array}\right] \left[ \begin{array}{c} \textbf{a}\\ \textbf{q}_1 \\ \textbf{q}_2 \end{array}\right] = \left[ \begin{array}{ccc} \textbf{I} &{}\quad -\alpha \textbf{B} &{}\quad \textbf{0} \\ \textbf{0} &{}\quad \textbf{I} &{}\quad -\textbf{I} \\ \textbf{0} &{}\quad \textbf{0} &{}\quad \textbf{I} \end{array}\right] \left[ \begin{array}{c} \textbf{f}_{0}\\ \textbf{f}_{1} \\ \textbf{f}_{2} \end{array}\right] . \end{aligned}$$

These equations can be rewritten in terms of the block matrices

77 $${\textsf {\bf M}} '{{\ddot{\textsf {\bf q}}}}+{\textsf {\bf C}}'{{\dot{\textsf {\bf q}}}}+ {\textsf {\bf K}}'{\textsf {\bf q}}= {{\textsf {\bf P}}} {\textsf {\bf f}}.$$

From now on we use sanserif boldface letters for block matrices. Here ${\textsf {\bf{q}}}$ is the displacement matrix

78 $$\begin{aligned} {{{{\textsf {{{\bf q}}}}}}}=\left[ \begin{array}{c} \textbf{a}\\ \textbf{q}_1 \\ \textbf{q}_2 \end{array}\right] , \end{aligned}$$

where $\textbf{a}_{5\times 1}$ is the antenna’s mode amplitude, $\textbf{q}_{1\, 6\times 1}$ and $\textbf{q}_{2\,6\times 1}$ are vectors of the relative displacements for the first and second resonators of each transducer. The mass matrix is

79 $$\begin{aligned} {{{{\textsf {{{\bf M}}}}}}}'=\left[ \begin{array}{ccc} M_S\,\textbf{I} &{}\quad \textbf{0} &{}\quad \textbf{0} \\ M_1\alpha \textbf{B}^T &{}\quad M_1\textbf{I} &{}\quad \textbf{0} \\ M_2\alpha \textbf{B}^T &{}\quad M_2\textbf{I} &{}\quad M_2\textbf{I} \end{array}\right] , \end{aligned}$$

where $\textbf{B}_{5\times 6}$ is the model matrix. Let us rewrite this matrix in term of the effective mass using the mass ratios $\mu ^2$ and $\nu ^2$ given in Eq. (68). We have

80 $$\begin{aligned} {{{{\textsf {{{\bf  M}}}}}}}'=M_{\text{eff}}\left[ \begin{array}{ccc} \nu ^2\,\textbf{I} &{}\quad \textbf{0} &{}\quad \textbf{0} \\ \mu ^2\alpha \textbf{B}^T &{}\quad \mu ^2\textbf{I} &{}\quad \textbf{0} \\ \mu ^4\alpha \textbf{B}^T &{}\quad \mu ^4\textbf{I} &{}\quad \mu ^4\textbf{I} \end{array}\right] =M_{\text{eff}}{{{{\textsf {{{\bf M}}}}}}}. \end{aligned}$$

The stiffness matrix is

81 $$\begin{aligned} {{{{\textsf {{{\bf K}}}}}}}'=\left[ \begin{array}{ccc} \text{Diag}({\kappa _m}_S) &{}\quad -\kappa _1\alpha \textbf{B} &{}\quad \textbf{0} \\ \textbf{0} &{}\quad \kappa _1\textbf{I} &{}\quad -\kappa _2\textbf{I} \\ \textbf{0} &{}\quad \textbf{0} &{}\quad \kappa _2\textbf{I} \end{array}\right] \end{aligned}$$

and using $\kappa _{mS}=M_Sw_m^2$, $\kappa _1=M_1w_0^2$, $\kappa _2=M_2w_0^2$ and the mass ratios $\mu ^2$ and $\nu ^2$ it reads

82 $$\begin{aligned} {{{{\textsf {{{\bf K}}}}}}}'=M_{\text{eff}}w_0^2\left[ \begin{array}{ccc} \nu ^2\text{Diag}\left( \frac{w_m^2}{w_0^2}\right) &{}\quad -\mu ^2\alpha \textbf{B} &{}\quad \textbf{0} \\ \textbf{0} &{}\quad \mu ^2\textbf{I} &{}\quad -\mu ^4\textbf{I} \\ \textbf{0} &{}\quad \textbf{0} &{}\quad \mu ^4\textbf{I} \end{array}\right] =M_{\text{eff}}w_0^2{{{{\textsf {{{\bf K}}}}}}} \end{aligned}$$

The damping matrix is

83 $$\begin{aligned} {{{{\textsf {{{\bf C}}}}}}}'=\left[ \begin{array}{ccc} \text{Diag}({C_m}_S) &{}\quad -C_1\alpha \textbf{B} &{}\quad \textbf{0} \\ \textbf{0} &{}\quad C_1\textbf{I} &{}\quad -C_2\textbf{I} \\ \textbf{0} &{}\quad \textbf{0} &{}\quad C_2\textbf{I} \end{array}\right] . \end{aligned}$$

and using ${C_m}_S=M_S w_m/Q_m$, $C_1=M_1w_0/Q_1$, $C_2=M_2w_0/Q_2$ and the mass ratios $\mu ^2$ and $\nu ^2$ it reads

84 $$\begin{aligned} {{{{\textsf {{{\bf C}}}}}}}'=M_{{eff}}\frac{w_0}{Q}\left[ \begin{array}{ccc} \nu ^2\text{Diag}\left( \frac{w_mQ}{w_0Q_m}\right) &{}\quad -\mu ^2\frac{Q}{Q_1}\alpha \textbf{B} &{}\quad \textbf{0} \\ \textbf{0} &{}\quad \mu ^2\frac{Q}{Q_1}{} \textbf{I} &{}\quad -\mu ^4\frac{Q}{Q_2}{} \textbf{I} \\ \textbf{0} &{}\quad \textbf{0} &{}\quad \mu ^4\frac{Q}{Q_2}{} \textbf{I} \end{array}\right] =M_{\text{eff}}\frac{w_0}{Q}{{{{\textsf {{{\bf C}}}}}}}. \end{aligned}$$

where $Q_m$, $Q_1$ and $Q_2$ are respectively the quality factor of the modes of the sphere, the first and second resonators. In our case $Q_m=Q$ where *Q* is the measured value of the quality factor of the sphere, but we leave at it is for generality. The movement equation then reads

85 $$\begin{aligned} M_{\text{eff}}{{{{\textsf {{{\bf M}}}}}}}{{\ddot{{{{\textsf {{{\bf q}}}}}}}}}+ M_{\text{eff}}\frac{w_0}{Q}{{{{\textsf {{{\bf K}}}}}}}{{\dot{{{{\textsf {{{\bf q}}}}}}}}}+ M_{\text{eff}}w_0^2{{{{\textsf {{{\bf K}}}}}}}{{{{\textsf {{{\bf q}}}}}}}={{{{\textsf {{{\bf P}}}}}}}{{{{\textsf {{{\bf f}}}}}}}. \end{aligned}$$

We will need to diagonalize the matrix ${{{{\textsf {{{\bf M}}}}}}}^{-1}{{{{\textsf {{{\bf K}}}}}}}$, but this matrix is not symmetric. In order to symmetrize it we change the coordinates defining ${{{{\textsf {{{\bf q}}}}}}}={{{{\textsf {{{\bf N}}}}}}}{{{{\textsf {{{\bf y}}}}}}}$ where

86 $$\begin{aligned} {{{{\textsf {{{\bf N}}}}}}}=\left[ \begin{array}{ccc} \textbf{I}/\nu &{}\quad \textbf{0} &{}\quad \textbf{0} \\ \textbf{0} &{}\quad \textbf{I}/\mu &{}\quad \textbf{0} \\ \textbf{0} &{}\quad \textbf{0} &{}\quad \textbf{I}/\mu ^2 \end{array}\right] \end{aligned}$$

and pre-multiply by ${{{\textsf {{{\bf N}}}}}}$

87 $$\begin{aligned} M_{\text{eff}}{{{\textsf {{{\bf NMN}}}}}}\ddot{{{{\textsf {{{\bf y}}}}}}}+ M_{\text{eff}}\frac{w_0}{Q}{{{\textsf {{{\bf NKN}}}}}}\dot{{{{{\textsf {{{\bf y}}}}}}}}+ M_{\text{eff}}w_0^2{{{\textsf {{{\bf NKNy}}}}}}={{{\textsf {{{\bf NPf}}}}}}. \end{aligned}$$

Multiplying both sides of the equation by

88 $$\begin{aligned} {{{\textsf {{{\bf (NMN)}}}}}}^{-1} = \left({\begin{array}{*{20}l} \textbf{I} &{}\quad \textbf{0} &{}\quad \textbf{0}\\ -\gamma \textbf{B}^T &{}\quad \textbf{I} &{}\quad \textbf{0}\\ \textbf{0} &{}\quad -\mu &{}\quad \textbf{I} \end{array}}\right) \end{aligned}$$

and defining $2\beta =\frac{w_0}{Q}$ we get

89 $$\begin{aligned} M_{\text{eff}}{\ddot{{{{{\textsf {{{\bf y}}}}}}}}}+ 2\beta M_{\text{eff}}{{{\textsf {{{\bf (NMN)}}}}}}^{-1}{{{\textsf {{{\bf NCN}}}}}}\dot{{{{{\textsf {{{\bf y}}}}}}}}+ M_{\text{eff}}w_0^2{{{\textsf {{{\bf (NMN)}}}}}}^{-1}{{{\textsf {{{\bf NKNy}}}}}}= {{{\textsf {{{\bf (NMN)}}}}}}^{-1}{{{\textsf {{{\bf NPf}}}}}}. \end{aligned}$$

Let us define the variables ${{{{\textsf {{{\bf M}}}}}}}_y$, ${{{{\textsf {{{\bf K}}}}}}}_y$ and ${{{{\textsf {{{\bf P}}}}}}}_y$, where the subscript is the indicative that these matrices are of the equation for ${{{{\textsf {{{\bf y}}}}}}}$. The equation then reads

90 $$\begin{aligned} M_{\text{eff}} \left( {\ddot{{{{{\textsf {{{\bf y}}}}}}}}}+ 2\beta {{{\textsf {{{\bf C}}}}}}_y{\dot{{{{{\textsf {{{\bf y}}}}}}}}}+ w_0^2{{{\textsf {{{\bf K}}}}}}_y{{{\textsf {{{\bf y}}}}}} \right) ={{{{\textsf {{{\bf P}}}}}}}_y{{{\textsf {{{\bf f}}}}}}, \end{aligned}$$

whose Fourier transform is

91 $$\begin{aligned} { M_{\text{eff}} \left( -w^2{{{{\textsf {{{\bf I}}}}}}}+ 2\beta jw{{{\textsf {{{\bf C}}}}}}_y+ w_0^2{{{\textsf {{{\bf K}}}}}}_y \right) {{\tilde{{{{{\textsf {{{\bf y}}}}}}}}}}={{{{\textsf {{{\bf P}}}}}}}_y{{\tilde{{{{{\textsf {{{\bf f}}}}}}}}}}.} \end{aligned}$$

This can be rewritten

92 $$\begin{aligned} M_{\text{eff}}L_\text{BF}(w){\tilde{\textsf{\bf{y}}}}={\textsf{\bf{P}}}_{y}{\tilde{\textsf{\bf{f}}}} \end{aligned}$$

In case of brute force solution we invert for each *w* the matrix in the lhs of this equation, using ${{{\textsf {{{\bf q}}}}}}={{{\textsf {{{\bf Ny}}}}}}$ giving

93 $$\begin{aligned} {{{\textsf {{{\bf q}}}}}}(w)={{{\textsf {{{\bf G}}}}}}_\text{BF}(w){{\tilde{{{{{\textsf {{{\bf f}}}}}}}}}}(w) \end{aligned}$$

where ${{{\textsf {{{\bf G}}}}}}_\text{BF}(w)$ is the brute force transfer function of the input ${{\tilde{{{{{\textsf {{{\bf f}}}}}}}}}}$

94 $${{{\textsf {{{\bf G}}}}}}_\text{BF}(w)=\frac{1}{M_{\text{eff}}}{{{\textsf{{{\bf N}}}}}}L_\text{BF}^{-1}(w){{{{\textsf {{{\bf P}}}}}}}_y. $$

The matrices are

95 $$\begin{aligned} {{{{\textsf {{{\bf K}}}}}}}_y= & {{\textsf {{{\bf (NMN)}}}}}^{-1}{{{\textsf {{{\bf NKN}}}}}} = \left({\begin{array}{ccc}    {\text{diag}\frac{w_m^2}{w_0^2}}  & {-\gamma \textbf{B}}  & {\textbf{0}}   \\    {-\gamma \textbf{B}^T\text{diag}\frac{w_m^2}{w_0^2}}  & {{\frac{3\gamma ^2}{2\pi }{{\varvec{\Gamma }}}+\textbf{I}}}  & {-\mu \textbf{I}}\\    {\textbf{0}}  & {-\mu \textbf{I}}  & {(\mu ^2+1)\textbf{I}}\\   \end{array} } \right), \end{aligned}$$

96 $$\begin{aligned} {{{{\textsf {{{\bf C}}}}}}}_y= & {} {{{\textsf {{{\bf (NMN)}}}}}}^{-1}{{{\textsf {{{\bf NCN}}}}}} = \left({ {\begin{array}{*{20}l} \text{diag}\frac{w_mQ}{w_0Q_m} &{}\quad -\gamma \frac{Q}{Q_1}{} \textbf{B} &{}\quad \textbf{0}\\ -\gamma \textbf{B}^T\text{diag}\frac{w_mQ}{w_0Q_m} &{}\quad {\frac{3\gamma ^2}{2\pi }\frac{Q}{Q_1}{{\varvec{\Gamma }}}+\frac{Q}{Q_1}{} \textbf{I}} &{}\quad -\mu \frac{Q}{Q_2}{} \textbf{I}\\ \textbf{0} &{}\quad -\mu \frac{Q}{Q_1}{} \textbf{I} &{}\quad (\mu ^2+1)\frac{Q}{Q_2}{} \textbf{I} \end{array}}}\right) \end{aligned}$$

where $$\gamma =\alpha \mu /\nu $$ and

97 $$\begin{aligned} { {{\varvec{\Gamma }}}=\textbf{I}-\frac{1}{6}\textbf{1} } \end{aligned}$$

with $\textbf{1}$ a matrix full of ones. The matrix ${{{{\textsf {{{\bf P}}}}}}}_y$ is

98 $$\begin{aligned} {{{{\textsf {{{\bf P}}}}}}}_y={{{\textsf {{{\bf (NMN)}}}}}}^{-1}{{{\textsf {{{\bf NP}}}}}} =\left( {\begin{array}{*{20}l} \frac{1}{\nu }\textbf{I} &{}\quad -\frac{\gamma }{\mu }\textbf{B} &{}\quad \textbf{0}\\ -\frac{\gamma }{\nu }{} \textbf{B}^T &{}\quad {\frac{3\gamma ^2}{2\pi \mu }{{\varvec{\Gamma }}}+\frac{1}{\mu }\textbf{I}} &{}\quad -\frac{1}{\mu }\textbf{I}\\ \textbf{0} &{}\quad -\textbf{I} &{}\quad \left( 1+\frac{1}{\mu ^2}\right) \textbf{I} \end{array}}\right). \end{aligned}$$

We need to solve Eq. (91), to do this we have two tracks to follow: the first one is the traditional method of finding eigenvalues and eigenvectors to diagonalize the matrix in the lhs of this equation, the other is that we call brute force, we solve this equation inverting the matrix for each value of the angular frequency *w*. The first one is easier to find the eigen frequencies of the system. For the sensibility calculation we have done using both methods with identical results, the relative error is of the order of $10^{-7}$ in the degenerate case and of the order of $10^{-3}$ in non degenerate case.

For the solution using the traditional method, we need to do some approximations in the matrix ${{{{\textsf {{{\bf K}}}}}}}_y$ and in the matrix ${{{{\textsf {{{\bf C}}}}}}}_y$. First of all they must be symmetric and according to Caughey and O’Kelley in (1965)^58^, the general condition to uncouple the modal equations with $\varvec{M}$, $\varvec{K}$ and $\varvec{C}$ respectively as mass matrix, stiffness matrix and damping matrix is that $\varvec{K}\varvec{M}^{-1}\varvec{C}=\varvec{C}\varvec{M}^{-1}\varvec{K}$.

One reason that we can do approximations in the damping matrix is that we often have little information about the precise form for this matrix, so we are free to choose it in a way that simplifies the analysis. For this purpose, in our case, we make the approximations: from now on we use the measured value of the quality factor $Q=Q_m$, and given that

99 $$\begin{aligned} \text{Diag}\left( \frac{w_m}{w_0}\right) \approx \text{Diag}\left( \frac{w_m^2}{w_0^2}\right) \approx \textbf{I}, \end{aligned}$$

in the entries ${{{{\textsf {{{\bf K}}}}}}}_{y21}$ and ${{{{\textsf {{{\bf C}}}}}}}_{y21}$ we approximate $\text{Diag}\left( \frac{w_m}{w_0}\right) =\textbf{I}$, in the entry ${{{{\textsf {{{\bf C}}}}}}}_{y11}$ we approximate $\text{Diag}\left( \frac{w_m}{w_0}\right) =\text{Diag}\left( \frac{w_m^2}{w_0^2}\right)$ and use $Q=Q_1=Q_2$ in the entries ${{{{\textsf {{{\bf C}}}}}}}_{y12}$, ${{{{\textsf {{{\bf C}}}}}}}_{y22}$, ${{{{\textsf {{{\bf C}}}}}}}_{y23}$, ${{{{\textsf {{{\bf C}}}}}}}_{y32}$, ${{{{\textsf {{{\bf C}}}}}}}_{y33}$. So that we end with ${{{{\textsf {{{\bf C}}}}}}}_y={{{{\textsf {{{\bf K}}}}}}}_y$. Then we can use the same modal matrix ${{{{\textsf {{{\bf U}}}}}}}$ to diagonalize both matrices.

Let the matrix ${{{{\textsf {{{\bf U}}}}}}}$ diagonalize ${{{{\textsf {{{\bf K}}}}}}}_y$ and let us define ${{{{\textsf {{{\bf y}}}}}}}={{{{\textsf {{{\bf U}}}}}}}{{{{\textsf {{{\bf z}}}}}}}$ and pre-multiply by ${{{{\textsf {{{\bf U}}}}}}}^T$, then Eq. (91) reads

100 $$\begin{aligned} { M_{\text{eff}} \left( -w^2{{{{{\textsf {{{\bf U}}}}}}}^T{{{\textsf {{{\bf I}}}}}}{{{\textsf {{{\bf U}}}}}}}+ 2\beta jw{{{{\textsf {{{\bf U}}}}}}}^T{{{\textsf {{{\bf C}}}}}}_y{{{\textsf {{{\bf U}}}}}}+ w_0^2{{{{\textsf {{{\bf U}}}}}}}^T{{{\textsf {{{\bf K}}}}}}_y{{{\textsf {{{\bf U}}}}}} \right) {{\tilde{{{{\textsf {{{\bf z}}}}}}}}}}={{{\textsf {{{\bf U}}}}}}^T{{{{\textsf {{{\bf P}}}}}}}_y{{\tilde{{{{{\textsf {{{\bf f}}}}}}}}}}. \end{aligned}$$

As ${{{\textsf {{{\bf U}}}}}}$ diagonalize ${{{{\textsf {{{\bf K}}}}}}}_y$ and ${{{{\textsf {{{\bf D}}}}}}}$ being the diagonal matrix such that ${{{{\textsf {{{\bf D}}}}}}}={{{{\textsf {{{\bf U}}}}}}}^T{{{{\textsf {{{\bf K}}}}}}}_y{{{{\textsf {{{\bf U}}}}}}}$ this equation becomes

101 $$\begin{aligned} M_{\text{eff}} \left( -w^2{{{\textsf {{{\bf I}}}}}}+ 2\beta jw{{{\textsf {{{\bf D}}}}}}+ w_0^2{{{\textsf {{{\bf D}}}}}} \right) {{\tilde{{{{{\textsf {{{\bf z}}}}}}}}}}= {{{{\textsf {{{\bf U}}}}}}}^T{{{{\textsf {{{\bf P}}}}}}}_y{{\tilde{{{{{\textsf {{{\bf f}}}}}}}}}}, \end{aligned}$$

We omit the *w* dependence in some cases to leave the notation cleaner.

If we define the diagonal matrix

102 $$\begin{aligned} {{{{\textsf {{{\bf L}}}}}}}(w)= M_{\text{eff}} \left( -w^2{{{{\textsf {{{\bf I}}}}}}}+ 2\beta jw{{{{\textsf {{{\bf D}}}}}}}+ w_0^2{{{{\textsf {{{\bf D}}}}}}} \right) \end{aligned}$$

we get

103 $$\begin{aligned} {{{{\textsf {{{\bf L}}}}}}}(w){{\tilde{{{{{\textsf {{{\bf z}}}}}}}}}}(w)= {{{{\textsf {{{\bf U}}}}}}}^T{{{{\textsf {{{\bf P}}}}}}}_y{{\tilde{{{{{\textsf {{{\bf f}}}}}}}}}}(w) . \end{aligned}$$

We invert to find $\varvec{{\tilde{z}}}$

104 $$\begin{aligned} {{\tilde{{{{{\textsf {{{\bf z}}}}}}}}}}={{{{\textsf {{{\bf L}}}}}}}^{-1}(w){{{{\textsf {{{\bf U}}}}}}}^T {{{{\textsf {{{\bf P}}}}}}}_y{{\tilde{{{{{\textsf {{{\bf f}}}}}}}}}}, \end{aligned}$$

where

105 $$\begin{aligned} {{{{\textsf {{{\bf L}}}}}}}^{-1}(w)=\frac{1}{M_{\text{eff}}} \text{Diag}\left( \frac{1}{-w^2+(2j\beta w+w_0^2)D_{11}}, \cdots ,\frac{1}{-w^2+(2j\beta w+w_0^2)D_{1717}} \right) . \end{aligned}$$

Returning to the old variables we have

106 $$\begin{aligned} {{\tilde{{{{{\textsf {{{\bf q}}}}}}}}}}= {{{\textsf {{{\bf NU}}}}}}{} \textbf{L}^{-1}(w){{{{\textsf {{{\bf U}}}}}}}^T{{{{\textsf {{{\bf N}}}}}}}^{-1}{{{{\textsf {{{\bf M}}}}}}}^{-1} {{{{\textsf {{{\bf P}}}}}}}{{\tilde{{{{{\textsf {{{\bf f}}}}}}}}}}. \end{aligned}$$

The transfer functions for the input ${{\tilde{{{{{\textsf {{{\bf f}}}}}}}}}}$ will be

107 $$\begin{aligned} {{{{\textsf {{{\bf G}}}}}}}(w)={{{\textsf {{{\bf NU}}}}}}{{{\textsf {{{\bf L}}}}}}^{-1}(w){{{{\textsf {{{\bf U}}}}}}}^T {{{{\textsf {{{\bf N}}}}}}}^{-1}{{{{\textsf {{{\bf M}}}}}}}^{-1}{{{{\textsf {{{\bf P}}}}}}}, \end{aligned}$$

where the block matrix ${{{{\textsf {{{\bf G}}}}}}}$ can be written as

108 $$\begin{aligned} {{{{\textsf {{{\bf G}}}}}}}=\left({\begin{array}{*{20}l} \textbf{G}_{00} &{}\quad \textbf{G}_{01} &{}\quad \textbf{G}_{02} \\ \textbf{G}_{10} &{}\quad \textbf{G}_{11} &{}\quad \textbf{G}_{12} \\ \textbf{G}_{20} &{}\quad \textbf{G}_{21} &{}\quad \textbf{G}_{22} \end{array}}\right). \end{aligned}$$

Then, we can write Eq. (106) as

109 $$\begin{aligned} \left({\begin{array}{*{20}l} {{\tilde {\mathbf{a}}}} \\ {{\tilde{\textbf{q}}}}_1 \\ {{\tilde{\textbf{q}}}}_2 \end{array}}\right)=\left( {\begin{array}{*{20}l} \textbf{G}_{00} &{}\quad \textbf{G}_{01} &{}\quad \textbf{G}_{02} \\ \textbf{G}_{10} &{}\quad \textbf{G}_{11} &{}\quad \textbf{G}_{12} \\ \textbf{G}_{20} &{}\quad \textbf{G}_{21} &{}\quad \textbf{G}_{22} \end{array}}\right) \left({\begin{array}{*{20}l} {{\tilde{\textbf{f}}}}_0 \\ {{\tilde{\textbf{f}}}}_1\\ {{\tilde{\textbf{f}}}}_2 \end{array}}\right). \end{aligned}$$

### Classical noise power spectrum matrix

In this work we will assume that the noise is an ergodic wide sense stationary stochastic process being analysed in an interval of time $T_o$. Let *x*(*t*) with Fourier transform ${\tilde{x}}(w)$ be a process satisfying these conditions, then the Power Spectral Density (PSD) of *x* is calculated as (see Whalen Chap.(2)^59^ and Maggiore^49^ for details)

110 $$\begin{aligned} S_{xx}=E[{{\tilde{x}}}(w){\tilde{x}}(w)^*]T_o. \end{aligned}$$

Our system is contaminated with forces of thermal noise ${\varvec{f}}_\text{th}$, forces of back action on the membrane ${{\varvec{f}}}_\text{bk}$, series forces ${\varvec{f}}_\text{se}$ and phase forces ${\varvec{f}}_\text{ph}$. The measured quantity is the output $\textbf{q}_2$ (transducer membrane) of our system

111 $$\begin{aligned} {{\tilde{\textbf{q}}}}_2=\textbf{G}_{20}{{\tilde{\textbf{f}}}}_0+ \textbf{G}_{21}{{\tilde{\textbf{f}}}}_1+ \textbf{G}_{22}{{\tilde{\textbf{f}}}}'_2+ \textbf{G}_{22}{{\tilde{\textbf{f}}}}_{bk}+ {{\tilde{\textbf{f}}}}_{se}+{{\tilde{\textbf{f}}}}_{ph}. \end{aligned}$$

We have splited the forces on the sencond resonator ${{\tilde{\textbf{f}}}}_2={{\tilde{\textbf{f}}}}'_2+{{\tilde{\textbf{f}}}}_{bk}$. The PSD of the output $\textbf{q}_2$ is, assuming that the noise forces of different kind are non correlated and the forces ${\tilde{\textbf{f}}}$ are of thermal origin

112 $$\begin{aligned} {{{{\textsf {{{\bf S}}}}}}}_{qq}&= \textbf{G}_{20}E\big [{{\tilde{\textbf{f}}}}_0 {{\tilde{\textbf{f}}}}_0^\dagger \big ]\textbf{G}_{20}^\dagger + \textbf{G}_{21}E \big [{{\tilde{\textbf{f}}}}_1 {{\tilde{\textbf{f}}}}_1^\dagger \big ]\textbf{G}_{21}^\dagger + \textbf{G}_{22}E \big [{{\tilde{\textbf{f}}}}'_2 {{\tilde{\textbf{f}}}}{'\dagger }_2 \big ]\textbf{G}_{22}^\dagger + \textbf{G}_{22}E \big [{{\tilde{\textbf{f}}}}_{bk} {{\tilde{\textbf{f}}}}_{bk}^\dagger \big ]\textbf{G}_{22}^\dagger + E \big [{{\tilde{\textbf{f}}}}_{se}{{\tilde{\textbf{f}}}}_{se}^\dagger \big ]+ E \big [{{\tilde{\textbf{f}}}}_{ph}{{\tilde{\textbf{f}}}}_{ph}^\dagger \big ]\nonumber \\&= \textbf{G}_{20}{} \textbf{S}_{f_0f_0}{} \textbf{G}_{20}^\dagger + \textbf{G}_{21}{} \textbf{S}_{f_1f_1}{} \textbf{G}_{21}^\dagger + \textbf{G}_{22}\textbf{S}_{f'_2f'_2}{} \textbf{G}_{22}^\dagger + \textbf{G}_{22}{} \textbf{S}_{bk}\textbf{G}_{22}^\dagger + \textbf{S}_{se}+\textbf{S}_{ph}. \end{aligned}$$

The thermal noise power spectrum is based on the fluctuation dissipation theorem that stays that given a system with equation

113 $$\begin{aligned} {{{{\textsf {{{\bf L}}}}}}}(w){{\tilde{{{{{\textsf {{{\bf z}}}}}}}}}}={{\tilde{{{{{\textsf {{{\bf f}}}}}}}}}} \end{aligned}$$

the power spectrum of the fluctuation force ${{{{\textsf {{{\bf f}}}}}}}$ is given by114where  is the impedance of the system given by115In our case we have

116 $$\begin{aligned} {{{{\textsf {{{\bf S}}}}}}}_{th}=4k_BT\text{Re} \left[ \frac{{{{{\textsf {{{\bf L}}}}}}}(w)}{jw}\right] . \end{aligned}$$

But from Eqs. (77) and (83) we have

117 $$\begin{aligned} {{{{\textsf {{{\bf S}}}}}}}_{th}= 4k_BT{{{{\textsf {{{\bf P}}}}}}}^{-1}{{{{\textsf {{{\bf C}}}}}}}'=4k_BT \left( {\begin{array}{*{20}l} M_S\frac{w_0}{Q}{} \textbf{I}_{5\times 5} &{} \textbf{0} &{} \textbf{0}\\ \textbf{0} &{} M_1\frac{w_0}{Q_1}{} \textbf{I}_{6\times 6} &{} \textbf{0}\\ \textbf{0} &{} \textbf{0} &{} M_2\frac{w_0}{Q_1}{} \textbf{I}_{6\times 6} \end{array}} \right) = \left( {\begin{array}{*{20}l} \textbf{S}_{f_0f_0} &{} \textbf{0} &{} \textbf{0}\\ \textbf{0} &{} \textbf{S}_{f_1f_1} &{} \textbf{0}\\ \textbf{0} &{} \textbf{0} &{} \textbf{S}_{f_2f_2} \end{array}} \right) \qquad [\text{N}^2/\text{Hz}]. \end{aligned}$$

The back action noise force acting on the membrane is^60^

118 $$\begin{aligned} \textbf{S}_{bk}=\frac{P^2_\text{inc}S_\text{a}}{2w_p^2} \left( \frac{2Q_e}{f_p}\frac{df}{dx}\right) ^2 \textbf{I}_{6\times 6} \qquad [\text{N}^2/\text{Hz}], \end{aligned}$$

where ${P_\text{inc}}$ is the pump oscillator power, ${S_\text{a}}$ is the amplitude noise spectral density of the pump oscillator, ${f_\text{p}}$ is the pump oscillator frequency, ${Q_\text{e}}$ is the transducer cavity electric Q, and *dx* is the membrane displacement.

The series noise acting directly on the output is

119 $$\begin{aligned} \textbf{S}_{se}=\frac{(T_\text{amp}+T)k_B}{P_\text{inc}} \left( \frac{2Q_e}{f_p}\frac{df}{dx}\right) ^{-2} \textbf{I}_{6\times 6} \qquad [\mathrm{m^2/Hz}] \end{aligned}$$

where ${T_\text{amp}}$ is the amplifier noise temperature, *T* is the thermodynamic temperature, and ${k_\text{B}}$ is the Boltzmann constant.

The phase noise also acting directly on the output is

120 $$\begin{aligned} \textbf{S}_{ph}=S_{p} \left( \frac{2\pi }{w}\frac{df}{dx}\right) ^{-2} \textbf{I}_{6\times 6} \qquad [\mathrm{m^2/Hz}]. \end{aligned}$$

where ${S_\text{p}}$ is the phase noise spectral density.

### Standard quantum limit noise

In the following section we will derive the expression of the standard quantum noise. This will allow us to obtain the standard quantum limit of the Schenberg detector. The power signal-to-noise ratio $\rho ^2$ for an optimum filter (matched filter) is^61^

121 $$\begin{aligned} \rho ^2=\frac{1}{2\pi }\int _{-\infty }^{\infty }\frac{|M(w)|^2}{S_{nn}^{{ds}}(w)}dw, \end{aligned}$$

where *M*(*w*) is the Fourier transform of the signal of interest and $S_{nn}^\text{ds}(w)$ the double side power spectral density of the noise. Our signal is the vector with the spherical amplitudes $\textbf{h}(t)$. Using the single side power spectral density matrix $\textbf{S}_{nn}(w)$, the expression of the power signal-to-noise ratio becomes

122 $$\begin{aligned} \rho ^2=\frac{4}{2\pi }\int _0^{\infty } {{\tilde{\textbf{h}}}}^\dagger (w){{{{\textsf {{{\bf S}}}}}}}_{nn}^{-1}(w) {{\tilde{\textbf{h}}}}(w)dw. \end{aligned}$$

For bursts of duration $\tau _g\approx 1\,\mathrm ms$ the maximum bandwidth frequency is $\Delta f_\text{max}\approx 1\,\mathrm kHz$ and ${{\tilde{\textbf{h}}}}(w)$ does not change very much from its value at the resonant frequency $f_0$ in the band $\Delta f$ of the detector. We can define a mean power spectral density ${\bar{S}}_{nn}$ such that this integral can be approximated by

123 $$\begin{aligned} \rho ^2= \frac{4\Delta w}{2\pi }|{{\tilde{\textbf{h}}}}^\dagger (w_0)| \left( \frac{1}{\Delta w}\int _0^{\infty } {\hat{{\tilde{\textbf{h}}}}}^\dagger {{{{\textsf {{{\bf S}}}}}}}_{nn}^{-1}(w) {\hat{{\tilde{\textbf{h}}}}}dw \right) |{{\tilde{\textbf{h}}}}(w_0)| = \frac{4|{{\tilde{\textbf{h}}}}(w_0)|^2\Delta f}{{\bar{S}}_{nn}}, \end{aligned}$$

where $$\textbf{h}(w_0)=|\textbf{h}(w_0)|{\hat{{\tilde{\textbf{h}}}}}$$,

124 $$\begin{aligned} |\textbf{h}(w_0)|=\sqrt{\sum _{m=-2}^2h_m^2(w_0)} \end{aligned}$$

and

125 $$\begin{aligned} {\bar{S}}_{nn}=\left( \frac{1}{\Delta w}\int _0^{\infty } {\hat{{\tilde{\textbf{h}}}}}^\dagger {{{{\textsf {{{\bf S}}}}}}}_{nn}^{-1}(w) {\hat{{\tilde{\textbf{h}}}}}dw \right) ^{-1}. \end{aligned}$$

We can obtain $|\textbf{h}(w_0)|$ as a function of the energy deposited by the burst on the sphere using the formula^62^

126 $$\begin{aligned} E_s=\frac{1}{2M}\left| \int _{-\infty }^{\infty }f(t)\text{e}^{jw_0 t}dt\right| ^2, \end{aligned}$$

where *f*(*t*) is the external force acting on the harmonic oscillator and *M* its mass. Starting from the movement equation for the sphere modes (Eq. 31), the mass of the mode is $M_S$ as a result of the normalization condition and the force is $f(t)=\frac{1}{2}M_S\chi R\ddot{h}_m(t)$. The integration gives for each mode *m*

127 $$\begin{aligned} E_{sm}=\frac{1}{8}M_S\chi ^2R^2w_0^4 \left| {\tilde{h}}_m(w_0) \right| ^2, \end{aligned}$$

while the energy deposited in all modes is

128 $$\begin{aligned} E_s=\frac{1}{8}M_S\chi ^2R^2w_0^4 \left| {{\tilde{\varvec{h}}}}(w_0) \right| ^2. \end{aligned}$$

The sensitivity is obtained when $\rho ^2=1$. With this value, comparing Eq. (123) with Eq. (128) we obtain the mean power density spectrum as a function of the energy deposited in the sphere

129 $$\begin{aligned} {\bar{S}}_{nn}=\frac{32E_s\Delta f}{M_S\chi ^2 R^2w_0^4}. \end{aligned}$$

The energy deposited in terms of the number of phonons *n* is $E_s=n\hbar w_0$. The sensitivity at the quantum limit is when $n=1$

130 $$\begin{aligned} S_\text{SQL}=\frac{32\hbar \Delta f}{M_S\chi ^2 R^2w_0^3}. \end{aligned}$$

We can write this expression as a function of the longitudinal sound velocity and the longitudinal wave vector for the quadrupolar mode $w_0=w_{12}=q_{12}c_{l}$

131 $$\begin{aligned} S_\text{SQL}=\frac{32\hbar \Delta f}{M_S\chi ^2(q_{12}R)^2c_{l}^2w_0}. \end{aligned}$$

For Schenberg at $4\,\mathrm K$, $f_0=f_{12}=3205.94\,\mathrm{Hz}$, $M_S=1124\,\mathrm{kg}$, $\chi =-0.6004$, $R=32.214\,\mathrm{cm}$ and $\Delta f=110\,\mathrm{Hz}$. With these values the spectral amplitude is

132 $$\begin{aligned} h_S(w_0)=\sqrt{S_\text{SQL}}=3.29\times 10^{-23}\,\sqrt{{\text{Hz}}^{-1}}. \end{aligned}$$

### Sensitivity for classical noise

The spectral amplitude $h_S(w)$ represents the input GW spectrum that would produce a signal equal to the noise spectrum observed at the output of the antenna instrumentation.

A useful way to characterize the sensitivity of a GW detector is to calculate the $h_S(w)$ such that with optimal filtering the signal to noise ratio

133 $$\begin{aligned} \rho ^2=\frac{1}{2\pi }\int _{-\infty }^\infty \sigma (w)dw \end{aligned}$$

is equal to 1 for each bandwidth. Here

134 $$\begin{aligned} \sigma (w)={{\tilde{\varvec{q}}}}_2^\dagger {\textbf{S}}^{-1}_{qq}{{\tilde{\varvec{q}}}}_2, \end{aligned}$$

where $\varvec{q}_2$ are the output of the second transducer’s resonators, $\dagger$ stands for Hermitean conjugate. The sensitivity of the detector is obtained by searching for an input GW with amplitude $\textbf{h}$ that mimics the thermal noise at the output, with $\rho = 1$ per bandwidth. In other words we search for an $\textbf{h}$ such that

135 $$\begin{aligned} {{\tilde{\varvec{q}}}}_{2}^{\dagger }{} \textbf{S}_{qq}^{-1} {{{\tilde{\varvec{q}}}}_2}=1 \end{aligned}$$

or

136 $$\begin{aligned} X(w)^2 {{\tilde{\textbf{h}}}}^\dagger \mathbf{T_v}^T \textbf{G}_{20}^\dagger (w)\textbf{S}_{qq}^{-1} \textbf{G}_{20}(w)\mathbf{T_v}{{\tilde{\textbf{h}}}}=1. \end{aligned}$$

As we do not know the polarization neither the direction of the incoming wave we take the mean over all angles

137 $$\begin{aligned} \int _{\psi =0}^\pi \int _{\phi =0}^{2\pi }\int _{\theta =0}^\pi {{\tilde{\mathbf{h}}}}^\dagger \mathbf{T_v}^T \textbf{G}_{20}^\dagger (w)\textbf{S}_{qq}^{-1} \textbf{G}_{20}(w)\mathbf{T_v}{{\tilde{\textbf{h}}}} \frac{\sin \theta d\psi d\theta d\phi }{4\pi ^2}= \frac{1}{X(w)^2}. \end{aligned}$$

Then we obtain

138 $$\begin{aligned} \frac{1}{5}({{\tilde{h}}}_+^2+{{\tilde{h}}}_\times ^2) \text{Tr}(\textbf{G}_{20}^\dagger (w)\textbf{S}_{qq}^{-1} \textbf{G}_{20}(w)) =\frac{1}{X(w)^2} \end{aligned}$$

and the amplitude spectral density $h_S(w)=\sqrt{{{\tilde{h}}}_+^2+{{\tilde{h}}}_\times ^2}$

139 $$\begin{aligned} h_S(w)=\frac{\sqrt{5}}{\sqrt{X(w)^2 \text{Tr}(\textbf{G}_{20}^\dagger (w)\textbf{S}_{qq}^{-1} \textbf{G}_{20}(w))}}. \end{aligned}$$

**Figure 4:**
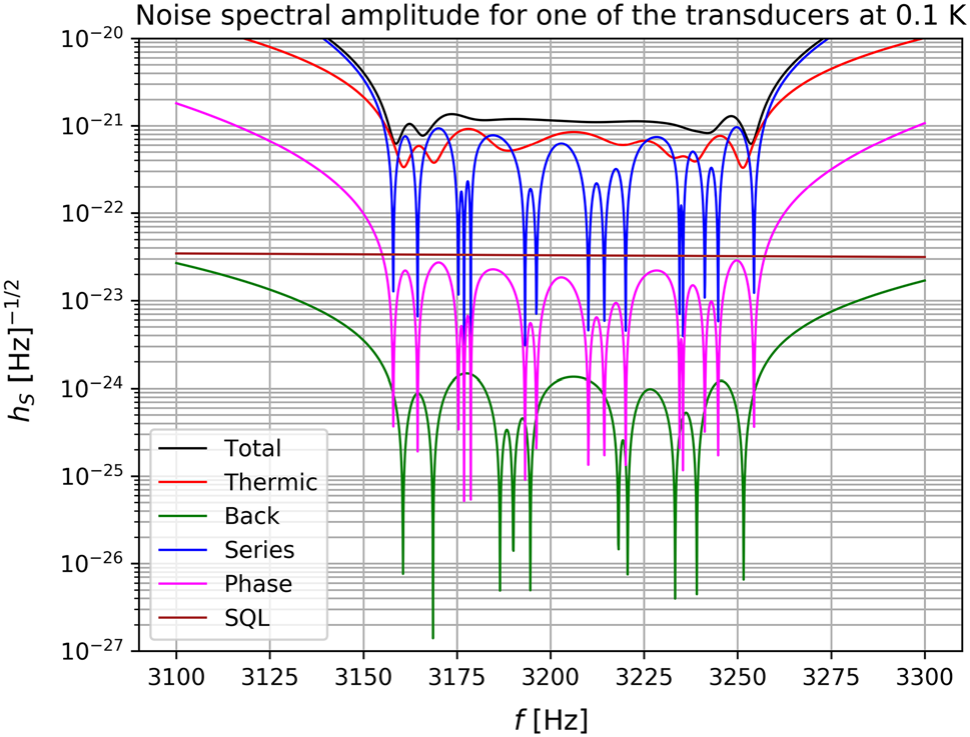
Sensitivity curves of the various type of noises for one of the six transducers of the Schenberg antenna at T = 0.1 K.

**Figure 5:**
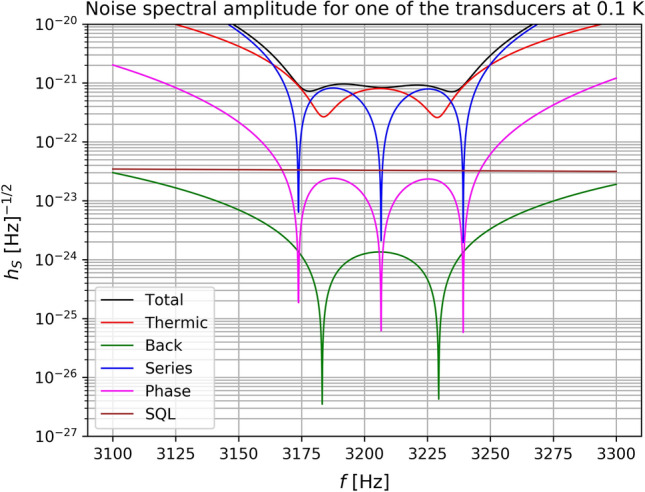
Sensitivity curves of the various type of noises for one of the six transducers of the Schenberg antenna system at T = 0.1 K for the sphere degenerated case.

The sensitivity curves for various kind of noises for each of the six transducers of the real antenna are shown in Fig. (4) using the parameters given in Table 3. In the case of a degenerated sphere the sensitivity curves for each of the six transducers would be as in Fig. 5.

**Table 3:** Parameters used in the sensitivity curve.

**Description**	**Value**
Thermodynamic Temperature	$T=100$ mK
Sphere mechanical Q	$Q=1\times 10^7$
Resonator 1 mechanical Q (transducer first mode)	$Q_1=1\times 10^6$
Resonator 2 mechanical Q (transducer second mode or membrane mode)	$Q_2=1\times 10^5$
Transducer central frequency	$F_T=3206.3$ Hz
Transducer minus frequency	$F_-=3172.5$ Hz
Transducer plus frequency	$F_+=3240.0$ Hz
Pump frequency	$F_\text{pump}=1\times 10^{10}$ Hz
Electric coupling constant	$\beta _\text{e}=0.65$
Frequency shift due to the displacement of the transducer membrane	$\frac{df}{dx}=7.26^{14}\,\text{Hz}/\text{m}$
Pump oscillator incident power	$P_\text{inc}=1\times 10^{-10}$ W
Amplifier noise temperature	$T_\text{amp}=10$ K
Electrical quality factor of the transducer cavity	$Q_\text{e}=3.8\times 10^5$
Phase noise spectral density	$S_\text{p}=1\times 10^{-13}$ dBc/$\text{Hz}$
Amplitude noise spectral density	$S_\text{a}=1\times 10^{-14}$ dBc/$\text{Hz}$
Loss in the microwave transmission line between transducer and amplifier	$L_\text{amp}=5$

The sensitivity of the Schenberg antenna will be better than the sensitivity of each transducer. Assuming that all transducers have the same sesitivity, the sensitivity of the Schenberg antenna ($h_S$) would be $(1/h_S)^2 = (1/h_{T1})^2 + (1/h_{T2})^2 + (1/h_{T3})^ 2 + (1/h_{T4})^2 + (1/h_{T5})^2 + (1/h_{T6})^2 = 6 \times (1/h_T)^2$, which implies that $h_S = h_T/ \sqrt{6}$.

## Discussions and conclusions

The calculation of the Schenberg antenna design sensitivity for each of the sphere six transducers was revised in this work taking into account both the degenerate (perfect sphere) and the non-degenerate sphere (quadrupole modes with their different frequencies), due to the symmetry break caused by the machining of the holes for the fixation of the transducers and the copper rod for the sphere suspension. As usual, all noises are referenced at the “input of the sphere” where the oscillating movement of the sphere surface occurs.

The dominant noises are the Brownian and the series noise, taking into account the parameters available for this initial version of the Schenberg antenna. For an advanced version of the Schenberg antenna (aSchenberg), which would reach the standard quantum limit of it ($3.29\times 10^{- 23} \text {Hz}^{-1/2}$), the sensitivity at each of the six transducers would be $\sqrt{6}$ times this or ($\sim 8\times 10^{- 23} \text {Hz}^{-1/2}$). To achieve this sensitivity at each niobium transducer we have to replace them with sapphire or silicon transducers, and with niobium coating in the microwave cavity region. In this way, we could reach mechanical quality factors of the order of $10^{8}$^63^. The sphere would have to undergo annealing or be replaced by another material, such as beryllium copper. Values of mechanical Qs close to $10^{8}$ have already been reached by Frossati (1996)^64^ for small copper-beryllium spheres.

Series noise can be minimized by rounding the edges of the transducer microwave klystron cavities, using a niobium deposition with less than 100 parts per million impurities, to increase the already achieved 380k electrical quality factor by a factor of 10 or more. The loss $L_\text{amp}$ in the microwave transmission line that carry the signal from the transducer to the cryogenic amplifier (the first line of amplifiers in the system) would need to be reduced by a factor of 5. This could be achieved using niobium coaxial cables. Finally, the electronics used in the cryogenic amplifiers would need to be replaced by one that would reduce the noise temperature from 10 K to 1 K, at the operating frequency of 10 GHz.

All these modifications, necessary to reach the standard quantum limit, are challenging, but not impossible to achieve for the small spherical antenna of 0.65 m in diameter. As parametric transducers are used, it would be possible to perform signal squeezing and exceeds the standard quantum limit, but this would require higher mechanical and electrical Qs and even less noisy electronics, which starts to be unfeasible or doubtful to be achieved.

Note, however, that the sensitivity achieved by aLIGO in the O3 run has already reached the standard quantum limit of this spherical antenna, therefore, the only reasonable justification for remounting the Schenberg antenna and trying to place it in the sensitivity of the standard quantum limit would be to detect gravitational waves using another physical principle, different from the one used by laser interferometers. This other physical principle would be the absorption of the gravitational wave energy by a resonant mass. The question that arises, then, is whether gravitational wave signals reach Earth with sufficient amplitude to be detected by the spherical antenna operating at the standard quantum limit. To answer this question, we are analyzing aLIGO’s O3 data in the range where the Schenberg antenna is most sensitive: 3.15 kHz to 3.26 kHz, looking for any type of signal (burst, chirp, continuous or stochastic). We look forward to providing the results of this investigation in the near future.

In addition, we would like to point out that the innovations in this work are the sensitivity calculation for the non-degenerate case, new relations for the model matrix **B**, and redefinition of the effective mass. Also was inovative to use the experimental values of the monopole and quadrupole mode frequencies at 2 K and 300 K in the determination of the elastic constant of the material and as a consequence the value of the transversal and longitudinal sound speed.

### Supplementary Information

Supplementary Information.

## Data Availability

this manuscript has no associated data or the data will not be deposited. All data generated during this study are contained in this published article.
