# Supplementary material for: The design strain sensitivity of the schenberg spherical resonant antenna for gravitational waves
Source: Sci Rep. 2023 Oct 17;13:17706. doi: 10.1038/s41598-023-43808-1 (PMC10582151; doi:10.1038/s41598-023-43808-1)
Supplement: Supplementary file 1 — Supplementary Information. [file 41598_2023_43808_MOESM1_ESM.pdf]

Appendix A EFFECTIVE MASS

The effective mass of the antenna that a transducer sees according to Zhou [65] is the mass that placed on the surface of the sphere acquires the same energy that the sphere has. Let us start calculating the equivalent mass for the quadrupole modes. The kinetic energy of the antenna for the quadrupole mode m is

$$E_k = \frac{1}{2}\int \rho \Psi_m^2 \dot{a}_m^2 d^3x = \frac{1}{2} M_S \dot{a}_m^2. \tag{140}$$

The velocity of the surface at the position of the transducer a in radial direction for the mode m is

$$v_{rma} = \dot{a}_m \mathbf{\Psi}_m \cdot \boldsymbol{e}_a = \dot{a}_m \alpha Y_m(\theta_a, \phi_a). \tag{141}$$

The kinetic energy for the effective mass of the mode m in this position is

$$E_k = \frac{1}{2} M_m \alpha^2 Y_m^2(\theta_a, \phi_a) \dot{a}_m^2. \tag{142}$$

Comparing (140) and (142) we have the equation for the effective mass for the mode m

$$M_S = M_m \alpha^2 Y_m^2(\theta_a, \phi_a) \tag{143}$$

and rearranging the equation we have

$$\frac{M_S}{\alpha^2 M_m} = Y_m^2(\theta_a, \phi_a). \tag{144}$$

The sum over all modes gives

$$\frac{M_S}{\alpha^2} \sum_{m=-2}^{2} \frac{1}{M_m} = \sum_{m=-2}^{2} Y_m^2(\theta_a, \phi_a). \tag{145}$$

The sum rule for spherical harmonics for the quadrupole gives

$$\sum_{m=-2}^{2} Y_m^2(\theta, \phi) = \frac{5}{4\pi}. \tag{146}$$

If we define the equivalent mass for the modes of the sphere as

$$\frac{1}{M_{\text{eq}}} = \frac{1}{M_1} + \frac{1}{M_2} + \frac{1}{M_3} + \frac{1}{M_4} + \frac{1}{M_5} \tag{147}$$

then

$$M_{\text{eq}} = \frac{4\pi}{5\alpha^2} M_S \tag{148}$$

and using $M_S = 1124$ kg and $\alpha^2 = 8.28584$ we have

$$M_{\text{eq}} = 0.30228 M_S = 340.934\,\text{kg}. \tag{149}$$

As we show explicitly in the next section the effective mass for N transducers considering five modes is given by

$$M_{\text{eff}} = \frac{5}{N} M_{\text{eq}} \quad \text{for six transducers} \quad M_{\text{eff}} = 284.111\,\text{kg}. \tag{150}$$

A Explicit effective mass calculation for N transducers

B For N=1

With the transducer at the position a we have

$$\frac{1}{2}M_S\sum_{m=-2}^{2}\dot{a}_m^2=\frac{1}{2}M_{\mathrm{eff}_1}v_a^2. \tag{151}$$

The velocity of the sphere surface at this position is

$$v_a=\alpha\sum_{m=1}^{5}\dot{a}_mY_m(\theta_a,\phi_a). \tag{152}$$

The square is given by

$$v_a^2=\alpha^2\sum_{m,n=1}^{5}\dot{a}_m\dot{a}_nY_m(\theta_a,\phi_a)Y_n(\theta_a,\phi_a) \tag{153}$$

and the mean over the angles is

$$\overline{v_a^2}=\alpha^2\sum_{m,n=1}^{5}\dot{a}_m\dot{a}_n\frac{1}{4\pi}\underbrace{\int Y_m(\theta,\phi)Y_n(\theta,\phi)\sin\theta d\theta}_{\delta_{mn}}=\frac{\alpha^2}{4\pi}\sum_{m=-2}^{2}\dot{a}_m^2. \tag{154}$$

The mean of both sides of Eq.(151) results

$$\frac{1}{2}M_S\sum_{m=-2}^{2}\dot{a}_m^2=\frac{1}{2}M_{\mathrm{eff}_1}\overline{v_a^2}=\frac{1}{2}M_{\mathrm{eff}_1}\frac{\alpha^2}{4\pi}\sum_{m=-2}^{2}\dot{a}_m^2, \tag{155}$$

such that

$$M_{\mathrm{eff}_1}=\frac{4\pi}{\alpha^2}M_S=\frac{5}{1}\frac{4\pi}{5\alpha^2}M_S=\frac{5}{1}M_{\mathrm{eq}}=1705\,\mathrm{kg}. \tag{156}$$

C For N transducers

If we have N transducers the kinetic energy is function of

$$\bar{v}_1^2+\bar{v}_2^2+\cdots+\bar{v}_N^2=N\frac{\alpha^2}{4\pi}\sum_{m=-2}^{2}\dot{a}_m^2 \tag{157}$$

such that

$$M_{\mathrm{eff_N}}=\frac{1}{N}\frac{4\pi}{\alpha^2}M_S=\frac{5}{N}\frac{4\pi}{5\alpha^2}M_S=\frac{5}{N}M_{\mathrm{eq}}. \tag{158}$$

D For six transducers in truncated icosahedron configuration

In matrix notation Eq.(151) can be written

$$\frac{1}{2}M_S\dot{\mathbf{a}}^T\dot{\mathbf{a}} = \frac{1}{2}M_{\rm eff}\dot{\mathbf{u}}^T\dot{\mathbf{u}} = \frac{1}{2}M_{\rm eff}\alpha^2\dot{\mathbf{a}}^T\mathbf{B}\mathbf{B}^T\dot{\mathbf{a}}. \tag{159}$$

The model matrix

$$\mathbf{B} = \sqrt{\frac{5}{4\pi}}\begin{pmatrix} \frac{3\varphi+2}{4\varphi+3} & -\frac{3\varphi+2}{4\varphi+3} & 0 & -\frac{1}{\varphi+2} & 0 & \frac{1}{\varphi+2} \\ -\frac{\varphi+1}{4\varphi+3} & \frac{\varphi+1}{4\varphi+3} & 0 & -\frac{\varphi+1}{\varphi+2} & 0 & \frac{\varphi+1}{\varphi+2} \\ -\frac{2\varphi+1}{4\varphi+3} & -\frac{2\varphi+1}{4\varphi+3} & -\frac{2\varphi+1}{4\varphi+3} & \frac{\varphi}{\varphi+2} & \frac{\varphi}{\varphi+2} & \frac{\varphi}{\varphi+2} \\ \frac{\varphi+1}{\sqrt{3}(4\varphi+3)} & \frac{\varphi+1}{\sqrt{3}(4\varphi+3)} & -\frac{2(\varphi+1)}{\sqrt{3}(4\varphi+3)} & -\frac{\varphi+1}{\sqrt{3}(\varphi+2)} & \frac{2(\varphi+1)}{\sqrt{3}(\varphi+2)} & -\frac{\varphi+1}{\sqrt{3}(\varphi+2)} \\ -\frac{3\varphi+2}{\sqrt{3}(4\varphi+3)} & -\frac{3\varphi+2}{\sqrt{3}(4\varphi+3)} & \frac{2(3\varphi+2)}{\sqrt{3}(4\varphi+3)} & -\frac{1}{\sqrt{3}(\varphi+2)} & \frac{2}{\sqrt{3}(\varphi+2)} & -\frac{1}{\sqrt{3}(\varphi+2)} \end{pmatrix} \tag{160}$$

has special properties obtained, or directly from the matrix $\boldsymbol{B}$ of with the help of spherical harmonics sum rules

$$\mathbf{B}\mathbf{B}^T = \frac{3}{2\pi}\mathbf{I} \qquad \mathbf{B}^T\mathbf{B} = \frac{3}{2\pi}\left(\mathbf{I} - \frac{1}{6}\mathbf{1}\right) = \frac{3}{2\pi}\mathbf{\Gamma} \qquad \mathbf{B1} = \mathbf{0}. \tag{161}$$

Furthermore, the Moore-Pensore pseudo inverse of $\mathbf{B}$, $\mathbf{B}^+$ is

$$\mathbf{B}^+ = \mathbf{B}^T(\mathbf{B}\mathbf{B}^T)^{-1} = \frac{2\pi}{3}\mathbf{B}^T \tag{162}$$

so that

$$\mathbf{B}^+\mathbf{B} = \mathbf{\Gamma}. \tag{163}$$

From Eq.(159) we have

$$\frac{1}{2}M_S\dot{\mathbf{a}}^T\dot{\mathbf{a}} = \frac{1}{2}M_{\rm eff}\frac{3\alpha^2}{2\pi}\dot{\mathbf{a}}^T\dot{\mathbf{a}} \tag{164}$$

then

$$M_{\rm eff} = \frac{2\pi}{3\alpha^2}M_S = \frac{5}{6}M_{\rm eq} = 284\,\text{kg}. \tag{165}$$

Appendix B MOVEMENT EQUATION IN TERMS OF SURFACE DEFORMATION

We have seen that the movement equation for the modes of a bare sphere under the action of N external forces of the type $\boldsymbol{f}_a = f_a\delta(\boldsymbol{x}-\boldsymbol{x}_a)\boldsymbol{e}_a$ at the positions $\boldsymbol{x}_a$ is given by

$$\ddot{\mathbf{a}} + 2\beta\dot{\mathbf{a}} + w_0^2\mathbf{a} = \frac{1}{M_S}\alpha\mathbf{B}\mathbf{f}. \tag{166}$$

Multiplying this equation by $\alpha\mathbf{B}^T$ we obtain it in terms of the sphere surface deformation $\boldsymbol{u}$

$$\ddot{\mathbf{u}} + 2\beta\dot{\mathbf{u}} + w_0^2\mathbf{u} = \frac{1}{M_S}\alpha^2\mathbf{B}^T\mathbf{B}\mathbf{f} \tag{167}$$

and using Eq.(161) results in

$$\ddot{\mathbf{u}} + 2\beta\dot{\mathbf{u}} + w_0^2\mathbf{u} = \frac{3\alpha^2}{2\pi M_S}\mathbf{\Gamma f} = \frac{1}{M_{\rm eff}}\mathbf{\Gamma f}. \tag{168}$$

Finally the movement equation for the deformation of the sphere surface at the position of transducers is

$$M_{\rm eff}\ddot{\mathbf{u}} + 2M_{\rm eff}\beta\dot{\mathbf{u}} + M_{\rm eff}w_0^2\mathbf{u} = \mathbf{\Gamma f}. \tag{169}$$

Appendix C REAL VECTOR SPHERICAL HARMONICS

The orthogonal real vector spherical harmonics are given by [66]

$$\boldsymbol{Y}^L_{\ell m}(\theta,\phi) = Y^{\mathcal{R}}_{\ell m}(\theta,\phi)\hat{\boldsymbol{r}} \tag{170}$$

$$\boldsymbol{Y}^E_{\ell m}(\theta,\phi) = \frac{1}{\sqrt{\ell(\ell+1)}} r\boldsymbol{\nabla} Y^{\mathcal{R}}_{\ell m}(\theta,\phi) \tag{171}$$

$$\boldsymbol{Y}^M_{\ell m}(\theta,\phi) = \hat{\boldsymbol{r}} \times \boldsymbol{Y}^E_{\ell m}(\theta,\phi), \tag{172}$$

where the real spherical harmonics $Y^{\mathcal{R}}_{\ell m}$ are given by the real and imaginary part of the traditional spherical harmonics, Eq.(6), [67]. The real vector spherical harmonics obey the normalization condition

$$\int \boldsymbol{Y}^A_N \cdot \boldsymbol{Y}^B_{N'} \sin\theta d\theta d\phi = \delta_{NN'}\delta_{AB}. \tag{173}$$

Appendix D TRANSFORMATION OF h_{ij} FROM WAVE FRAME TO LAB FRAME

The polarization tensor for a GW propagating in Z direction of the wave frame with polarizations h_+ and $h_\times$ is [49]

$$\mathbf{h}_{WF} = \begin{pmatrix} h_+ & h_\times & 0 \\ h_\times & -h_+ & 0 \\ 0 & 0 & 0 \end{pmatrix}. \tag{174}$$

Let the matrix $\mathbf{A}(\theta,\phi,\psi) = \mathbf{R}_z(\psi)\mathbf{R}_y(\theta)\mathbf{R}_z(\phi)$ rotates the lab reference frame to the direction (θ,ϕ,ψ) using Euler's-y convention. Any vector $\boldsymbol{v}$ can be rotated by this direction using the transpose of this matrix $\mathbf{A}^T(\theta,\phi,\psi) = \mathbf{R}^T_z(\phi)\mathbf{R}^T_y(\theta)\mathbf{R}^T_z(\psi)$. In the case GW we are not interested in ψ rotation because this only mixes the h_+, $h_\times$ polarizations. Without the ψ rotation the matrix $\mathbf{A}$ becomes

$$\mathbf{A} = \begin{pmatrix} \cos\theta\cos\phi & \cos\theta\sin\phi & -\sin\theta \\ -\sin\phi & \cos\phi & 0 \\ \sin\theta\cos\phi & \sin\theta\sin\phi & \cos\theta \end{pmatrix}. \tag{175}$$

If we have an incoming wave in the direction of the z axis of the lab frame, after a rotation to the direction (θ,ϕ) it is seen from the lab frame as

$$\mathbf{h}_{LF} = \mathbf{A}^T\mathbf{h}_{WF}\mathbf{A}. \tag{176}$$
